# Computational Estimates of Membrane Flow and Tension Gradient in Motile Cells

**DOI:** 10.1371/journal.pone.0084524

**Published:** 2014-01-17

**Authors:** Ben Fogelson, Alex Mogilner

**Affiliations:** Department of Neurobiology, Physiology and Behavior and Department of Mathematics, University of California, Davis, Davis, California, United States of America; Emory University/Georgia Insititute of Technology, United States of America

## Abstract

All parts of motile cells, including the plasma membrane, have to translocate in the direction of locomotion. Both directed intracellular membrane transport coupled with polarized endo- and exocytosis and fluid flow in the plane of the plasma membrane can contribute to this overall plasma membrane translocation. It remains unclear how strong a force is required to generate this flow. We numerically solve Stokes equations for the viscous membrane flow across a flat plasma membrane surface in the presence of transmembrane proteins attached to the cytoskeleton and find the membrane tension gradient associated with this flow. This gradient is sensitive to the size and density of the transmembrane proteins attached to the cytoskeleton and can become significant enough to slow down cell movement. We estimate the influence of intracellular membrane transport and actin growth and contraction on the tension gradient, and discuss possible ‘tank tread’ flow at ventral and dorsal surfaces.

## Introduction

The plasma membrane plays several crucial roles in cell life: separating the inside of the cell from the environment; serving as a scaffold for regulatory and structural proteins; and organizing cytoskeletal dynamics [1]. The plasma membrane's mechanical characteristics, such as flow [2], tension [1], [3] and curvature [4] are also important for cellular phenomena, especially for cell motility. Here we mathematically and computationally examine the mechanical effect that plasma membrane flow and the associated membrane tension have on motile cell behavior.

**Figure 1:**
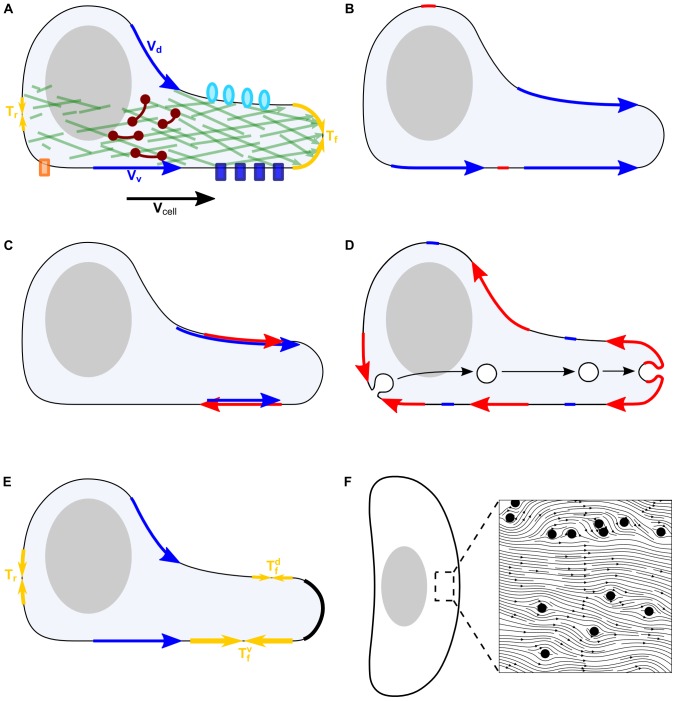
Possible types of membrane flow. A–E: View of the motile keratocyte cell's lamellipodium from the side. Shaded ellipsoid is the cell body. A: Growth of actin network (green) inside the lamellipodium pushes the leading edge forward, resisted by the membrane tension at the front (yellow arrows,*T_f_*). At the rear, membrane tension (yellow arrows, *T_r_*) pushes forward the disassembling actin networks. Besides the breaking actin network, breaking adhesions (orange rectangle) also resist rear retraction. Myosin (dark red dumbbells) powered contraction assists rear retraction. The membrane flows forward in the lab coordinate system (blue arrows) on the ventral and dorsal surfaces. Adhesions (blue rectangles) linked to the stationary actin network resist this flow at the ventral surface; transmembrane proteins (light blue ovals) resist this flow at the dorsal surface. B–E: blue (red) arrows show the membrane flow in the lab (moving cell) coordinate systems, respectively. B: One possibility is that the membrane flow is the same at the ventral and dorsal surfaces. In this case, these flows' rates are the same as the cell speed in the lab coordinate system, and the flows are zero in the cell frame. C: Example of tank-tread flow. D: In this case the membrane is transported from the rear to the front solely through the intracellular transport of membrane vesicles. The membrane flow is zero in the lab coordinate system and directed to the rear in the cell frame. E: Hypothesis about how the membrane flow can be the same on the ventral and dorsal surfaces for any different protein concentrations at these surfaces. This can be explained if the membrane flow across the leading edge (black) is obstructed. In this case, the membrane tension at the rear of the dorsal and ventral surfaces is the same, but rear-to-front gradients of tension are different along the ventral and dorsal surfaces because the same ventral and dorsal flows are resisted by different protein concentrations at these surfaces. Thus, tensions at the fronts of the ventral and dorsal surfaces are different. F: View of the motile keratocyte cell's lamellipodium from above. Shaded ellipsoid is the cell body. Insert: Cartoon of membrane flow around solid circular obstacles (proteins). Proteins attach to the cytoskeleton and/or the external environment, and so stay stationary (in the lab frame of reference). Thus, as the cell moves, the membrane is forced to flow around the proteins.

Cell migration on surfaces is a fundamental phenomenon underlying many physiological processes [5]. When a cell migrates, its parts, including its cytoskeleton, organelles, fluid cytoplasm and plasma membrane, have to translocate forward (Fig 1A). In many types of migrating cells, this forward translocation is driven by the dynamic actomyosin network, one of the main parts of the cytoskeleton, in which nascent actin filaments appear and grow at the cell front and push the leading edge forward, while older parts of the network disassemble and contract to pull the rear forward [6]. The actomyosin network adheres to the substrate via molecular complexes which contain integrins, and which span from actin to the substrate through the plasma membrane (Fig 1A). These adhesions are crucial for transducing the effective treadmill of the actomyosin array into forward propulsion of the cell [7]. The mechanisms by which organelles and cytoskeletal components move forward are not all entirely clear, but actomyosin contractions [8], microtubule-based motors [9] and membrane tension at the cell rear [10] contribute to these processes in various cells.

The plasma membrane enveloping the cell also has to translocate from the rear to the front. Because lipids and proteins in the mosaic membrane diffuse rapidly [11] in the membrane plane, the membrane can simply flow forward (Fig 1A). The flow of the plasma membrane can be supplemented, or even replaced, by directed intracellular movement of membrane vesicles mediated by motor-driven transport, so that endocytosis is responsible for removing plasma membrane at the rear and exocytosis for adding membrane at the front (Fig 1D). Indeed, in some cases there is evidence of polarized membrane trafficking [2], [12].

There is sometimes confusion in the literature that stems from the fact that the membrane flow looks different in the frame of the moving cell and in the lab coordinate system. In this paper, we will consider a cell steadily moving forward with the rate 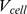 (Fig 1A), such as fish epithelial keratocyte [13]. We illustrate possible types of membrane flow in Fig 1B–D. The simplest possibility is if in the lab coordinate system both ventral and dorsal membranes flow forward (Fig 1B, blue arrows) at rates equal to the cell speed: 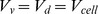. In the cell frame, there is no flow in this situation. Such a case was observed in a number of motile cells, including fibroblasts, fish keratocytes [14]–[16], leukocytes [17], and Dictyostelium amoebae [18]. A more complex possibility is a tank-tread flow in which, in the lab coordinate system, both ventral and dorsal membranes flow forward with different speeds; for example, the dorsal flow is faster (Fig 1C, blue arrows). Conservation of membrane material requires that in this case 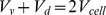. Then in the cell frame the dorsal flow 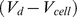 is directed forward, and the ventral flow 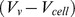 is directed rearward (Fig 1C, red arrows; note that 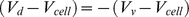). Finally, if intracellular traffic is solely responsible for forward membrane translocation, then in the lab coordinate system the membrane is stationary, while in the cell frame the membrane flows backward with equal rates at the ventral and dorsal surfaces equal to the cell speed 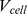 (Fig 1D, red arrows). Interestingly, in neuronal growth cones it was observed that membrane flow is directed from the front to the rear in the cell frame [19].

The membrane flow is determined by a force that drives it. This force arises from the gradient of the in-plane membrane tension [20] (Fig. 1A), so that the tension at the front, 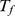, is higher than that at the rear, 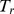, and so the more tensed membrane at the front pulls the plasma membrane forward against weaker tension at the rear. A front-rear membrane tension difference on the order of ∼1 pN/µm was indeed measured between the cell body and the growth cone in neurons [19], where this tension gradient was accompanied by membrane flow. For comparison, average membrane tension in different cell types varies widely, from a few pN/µm in neuronal growth cones to tens of pN/µm in melanoma cells [21] to hundreds of pN/µm in rapidly moving fish keratocytes [22].

Another set of factors determining the membrane flow are the mechanical properties of the membrane. With respect to out-of-plane deformations, the membrane has complex, partially elastic properties, but in-plane, the membrane is an incompressible viscous fluid [23]. Effective resistance to the flow of the membrane relative to the substrate arises largely due to transmembrane proteins, especially integrins, and proteins with domains which insert into or bind the membrane [24]. According to the mosaic model of plasma membrane structure, up to 50% of the membrane surface is occupied by such proteins, with lipid molecules filling the rest [25]. In general, lipids and membrane proteins are free to diffuse within the membrane, however, a subset of transmembrane proteins are restricted by binding to cytoskeletal structures [26], [27]. For example, membrane-associated proteins such as fodrin, plectin, and ankyrin attach to actin [28]. ERM and Ena/VASP are other examples of the protein families that associate both with actin and membrane. Given the abundance and strength of cell-substrate adhesion sites, they also contribute extensively to membrane-cytoskeleton interactions.

After a brief surge of interest in the role of membrane transport in cell locomotion [2], the efforts of motility researchers were largely concentrated on actin and myosin dynamics. The implicit assumption in most of the literature is that the plasma membrane flows forward effortlessly in motile cells. However, this assumption was never critically examined. Indeed, in the large part of the lamellipodium/lamellum (dynamic motile appendage of the cell, see Fig. 1), the actomyosin network is either almost stationary relative to the substrate [13] or undergoes rearward flow [29] in the direction opposite to that of cell migration. If a fraction of the transmembrane proteins are associated with this stationary or rearward moving cytoskeleton, then these proteins are effective buoys that obstruct the forward membrane flow. How much resistance these buoys exert on the flow is an open problem (an initial foray into this problem was recently made [24]). Recently, a few studies have reported that inhibition of membrane trafficking in several cell types, for example fibroblasts, endothelial cells and Dictyostelium, reduces persistent migration [30], [31]. One possible explanation is that membrane transport could be rate-limiting for cell forward translocation. Here, we compute the geometry of the plasma membrane flow and associated membrane tension gradients, and we predict conditions for high and low membrane tension gradients and for equal versus unequal ventral and dorsal flows.

## Results

### 1. Model

#### 1.1 Estimate for the drag coefficient of a single cylindrical protein

Variables and parameters in the models are defined and explained in Table 1 and Table 2. Saffman and Delbruck [32] derived an analytic estimate for the drag coefficient of a single cylindrical protein embedded in a flat, infinite sheet of membrane: 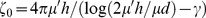
(1)


**Table 1:** Parameters in the models.

Variable notation	Value	Meaning	Reference
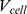	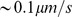	cell speed	[13]
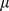	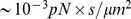	viscosity of aqueous medium	[32]
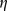	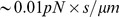	two-dimensional membrane viscosity	[51]
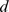	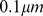	Adhesion complex diameter	[43]
	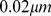	Transmembrane protein diameter	[44]
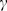	0.577	Euler-Mascheroni constant	[42]
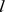	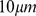	Order of magnitude ide-to-side lamellipodial width	[13]
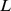	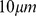	front-to-rear lamellipodial length	[13]
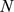	n/d, varies	number of transmembrane proteins linked to actin	NA
*φ*	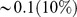	area fraction of the transmembrane proteins linked to actin	Assumed
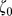	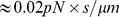	drag coefficient of a single cylindrical protein embedded in a flat, infinite sheet of membrane	Computed in this paper
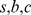	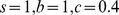	parameters defining the lamellipodial shape	Chosen in this paper to qualitatively fit the observed shape
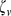 , 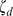 , 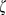	depends on other parameters	effective combined viscous drags of the transmembrane buoys in the ventral and dorsal surfaces	Computed in this paper
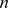	Defined in combination with 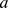 , 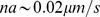	intracellular membrane vesicles per second per surface	[45], [46]
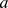	Defined in combination with 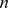 , 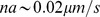	average membrane area per vesicle	[45], [46]
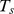	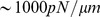	characteristic membrane tension that stalls protrusion	[22], [41]
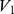	Defined in combination with other parameters	characteristic retraction speed of the cell rear, 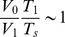	[10]
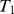	Defined in combination with other parameters	characteristic membrane tension at the rear, 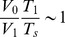	[10]

**Table 2:** Variables in the models.

Variable notation	Meaning	Dimension
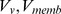	Speed of the membrane flow at the ventral surface	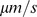
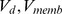	Speed of the membrane flow at the dorsal surface	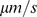
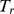	Membrane tension at the rear	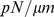
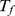	Membrane tension at the front	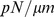
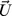	Local membrane flow rate	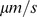
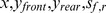	Coordinates describing lamellipodial boundary	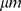
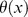	Angle between the direction of cell motion and the normal to the boundary	n/d

Here 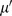 is the three-dimensional viscosity of the membrane, 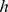 is the thickness of the membrane, 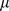 is the viscosity of the fluid surrounding the membrane, 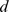 is the diameter of the protein, and 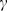 = 0.577 is the Euler-Mascheroni constant. Note that the two-dimensional membrane viscosity 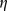 can be related to 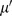 from equation (1) by the expression , so: 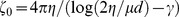
(2)


#### 1.2. Flow and tension distributions on the flat membrane surface

We model transmembrane proteins or protein complexes, such as adhesions, attached to the actin network as rigid cylindrical solid objects embedded in the membrane (Fig 1F). We consider steadily moving cells of a constant shape, like fish keratocytes (Fig 1F). In keratocytes, the motile appendage – the lamellipodium – is a flat actomyosin network with a characteristic canoe-like shape (Fig 1F). Most of this network, other than a small part at the very rear of the cell, is almost immobile relative to the surface [33], so we assume that in the lab coordinate system the transmembrane cylindrical ‘buoys’ are immobile. Following previous studies [24], [34]–[36], we represent the membrane as a thin layer of a continuous incompressible viscous fluid. Since the membrane is extremely thin, we treat it as a 2D fluid, and so we also treat the membrane domain of a protein as a circular obstruction in the 2D fluid domain. As the transmembrane buoys are held stationary during cell migration while the membrane advances forward in the lab coordinate system, the membrane and proteins interact mechanically to produce a non-uniform membrane flow, as sketched in Fig 1F. We model the flow as 2D viscous Stokes flow: 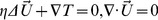
(3)


Here 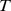 is the local membrane tension, and 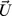 is the local membrane velocity in the lab coordinate system. Note that in two dimensions, the tension (which can be considered as pressure with opposite sign) has the dimensions Force/Length instead of the usual Force/Length^2^ in the 3D Stokes equations. Similarly, the membrane viscosity has the dimensions of Force × Time/Length, rather than the usual 3D viscosity dimensions of Force × Time/Length^2^. Another assumption we make is that we neglect attachment (between membrane and cytoskeleton) energy contribution to the membrane tension, so in equation (3) 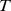 is the in-plane membrane tension. The length scale in this model is the characteristic length of the lamellipodium, 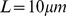, and the speed scale is the characteristic keratocyte speed 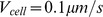 [13]. The natural membrane tension scale that we use in numerical calculations is 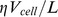.

#### 1.3. Cell shape, boundary conditions and protein distributions

**Figure 2:**
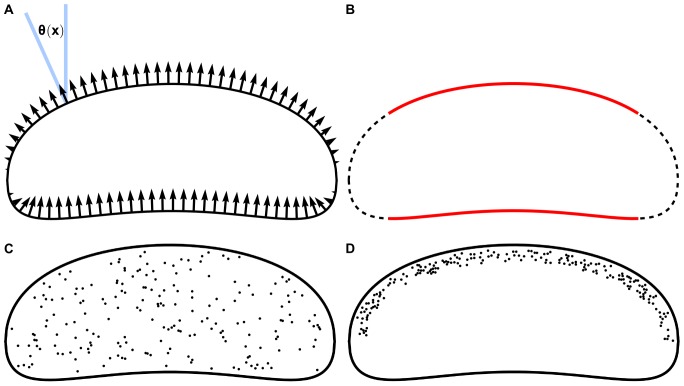
Lamellipodial shape and protein and velocity distributions. Shape of cell's lamellipodium given by equations (4–5). A: Sketch of velocity boundary condition (equation (6)) given by the graded radial extension model. Velocity at the boundary is normal to the boundary, and decreases in magnitude from a maximum when the normal points in the direction of cell motion to zero when the boundary is tangent to the direction of motion. B: Membrane boundary with leading and rear edges in red. We integrate tension over these red curves to compute the average tension at the front and rear of the cell, which allows us to compute the tension drop in the membrane. C–D: Randomly generated placements of transmembrane proteins distributed uniformly throughout the membrane (C) and distributed within 1 µm of the cell front (D).

We solve equation (3) for a flat membrane domain, which represents either the dorsal or ventral membrane surface. We define the shape of this domain similar to that of the keratocyte lamellipodium (Fig 2A) and represent the boundary of this domain by the equations: 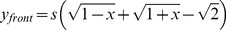
(4)for the front half of the boundary, and 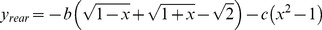
(5)for the rear half in the Cartesian coordinate system with the y-axis passing from the rear to the front through the middle of the cell, where 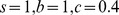. Here the length is non-dimensionalized using the lamellipodial length scale defined above.

We follow the Graded Radial Extension model for the steady lamellipodial locomotion [16] and require that at each point along the lamellipodial boundary the local membrane velocity is directed normal to the boundary, with magnitude 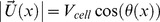
(6)where 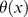 is the angle between the direction of cell motion and the normal to the boundary, as shown in Fig 2A. This boundary condition ensures that the plasma membrane translocates forward in a manner that preserves a steady cell shape. In non-dimensional units, 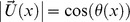.

We use the no-slip boundary condition 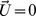 on the 2D circular surface of each protein. After the Stokes flow equation (3) with these boundary conditions is solved numerically (Methods), we calculate the average membrane tension at the front and rear edges of the cell (shown in Fig. 2B) as: 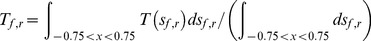
(7)where 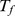 and 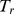 are the average membrane tensions at the front and rear edges, respectively, and 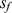 and 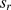 are the arclengths along the front and rear boundaries, 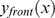 and 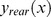, respectively.

We investigate the effect of transmembrane proteins or protein complexes on membrane tension by randomly placing circular obstructions in the interior of the membrane. We generate protein positions according to one of two distributions (Fig 2C,D). Proteins are either uniformly placed throughout the interior of the membrane (Fig 2C), or are restricted to being near the front of the cell (Fig 2D). We chose this latter distribution to account for the observations that at the ventral surface the adhesion complexes and respective traction are concentrated near the leading edge of the cell [37], [38] and that the membrane is loaded with proteins at the leading edge [39], [40]. For these front-loaded proteins, we required that each protein be entirely in the front half of the cell (defined by the line *y* = 0), and that the protein be within 1 µm of the front half of the cell boundary. For both the uniform and the front-loaded distributions, protein placement is generated in several steps: 1. Points are sampled from a uniform random distribution covering the entire cell. These points will become the centers for our circular proteins. 2. For each generated point, we check to ensure that it is in the correct region of space (either the cell interior for the uniform distribution or the front 1 µmof the cell for the front-loaded distribution). Any points failing this check are replaced by new random points. For numerical accuracy, we also require that all points be at least 0.25 µmaway from the membrane boundary. 3. To ensure that none of the circular proteins overlap with one another, we impose a short range repulsive interaction on neighboring points. This repulsion moves the protein centers until all centers are a distance of at least one diameter apart from one another.

#### 1.4. Tank-tread flow at ventral and dorsal surfaces

So far, we have described the membrane flow in the flat plane of either the ventral or the dorsal surface of the cell in the case when the ventral and dorsal flows are the same. The computational 2D model is useful for estimates of the membrane tension gradient and transmembrane protein resistance to the flow, and for visualizing the non-uniform membrane flow in the membrane plane. In the more general cases when either the flows are different or effects of actin mechanics and intracellular membrane transport are included, full simulation of the 2D flow would be difficult because of the complex actin mechanics and geometry of the curved plasma membrane at the rear and leading edges of the cell and unknown distribution of intracellular transport at the edges. However, simple estimates in the general cases can be made if we consider a simplified 1D model of the plasma membrane in the side view of the cell in the lab frame (Fig 1A). In this model, we approximate the flows in the ventral and dorsal planes as uniform (this approximation is in general very good, as shown in the results reported below) with rates 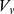 and 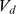, respectively. Let 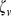 and 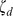 be the effective viscous resistance (due to the transmembrane proteins) of the ventral and dorsal surfaces, respectively. Then the membrane tension gradient between the front and rear of the cell is: 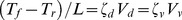
(8)for the dorsal and ventral surfaces. Note that coefficients 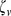 and 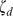 have different dimensions (Force × Time/Length^3^) than that of the protein viscous drag 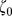. This can be best understood in the example of the linear approximation, when the drag imposed by 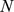 proteins is additive. In this case, if 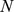 proteins, each characterized by the protein viscous drag 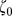, are distributed over the rectangular region of the membrane in the presence of the uniform flow with speed 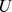, with the leading and rear edge length 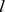 and the rear-to-front length 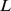, then the total resistance force from the proteins is equal to 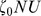, and the rear-to-front difference in membrane tension can be estimated is 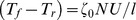. Introducing average density of proteins 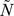, we can write: 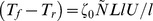, and 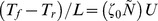. From comparison with equation (8), it is clear that coefficients 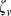 and 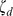 have dimension Force × Time/Length^3^ because the protein viscous drag 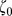 has dimensions Force × Time/Length, and the average density of proteins 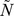 has dimensions 1/Length^2^.

We complement equations (8) with a third equation for conservation of membrane material for the plasma membrane: 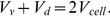
(9)


We will solve the three equations (8–9) for the three unknowns (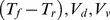) below.

#### 1.5. Effects of actin pushing at the front, actin contraction at the rear and intracellular membrane transport

Another similar 1D model considers the effect of intracellular membrane transport and force balances at the front and rear of the motile cell on both the membrane flow and the tension gradient. For simplicity, in this model we consider equal viscous resistance 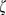 for the ventral and dorsal surfaces, and therefore equal respective membrane flows equal to 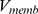 at these two surfaces. In this case, the force balance for the membrane flow and membrane tension gradient has the form: 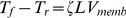
(10)


Let 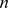 intracellular membrane vesicles with average membrane area 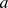 per vesicle per second per surface (dorsal or ventral) be transported from the rear to the front of the cell (Fig 1D) per one micron of the leading edge. Then the protrusion length 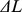 per time 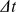 is equal to 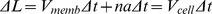. Therefore, the cell speed, 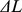/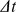, is equal to: 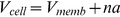
(11)


The two equations (10–11) for the four unknown variables (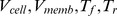) have to be complemented by the force balances at the front and rear of the cell. At the front, we use the force-velocity relation for the actin network polymerizing against the membrane load: 
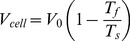
(12)


Here 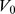 is the free polymerization rate in the absence of load, and the expression in brackets is the force-dependent factor that accounts for the slowing down of protrusion by membrane tension. Parameter 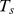 is the stall tension at which the protrusion halts. For simplicity we use the linearized force-velocity relation, which is an approximation to the non-linear relations measured and fitted for keratocytes [13], [41]. At the rear, we use the force-velocity relation for the disassembly-weakened actin network being crushed and hauled forward by the membrane tension, which was suggested and fitted to the data in [10]: 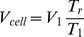
(13)


Here 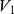 is the rate of the cell rear retraction generated when the membrane tension at the cell rear is equal to parameter 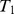. Below, we solve the four equations (10–13) analytically, allowing us to examine the effects of intracellular transport and actin mechanics on membrane flow and tension gradients.

### 2. 2D membrane flow and tension distributions in the plane of the membrane

**Figure 3:**
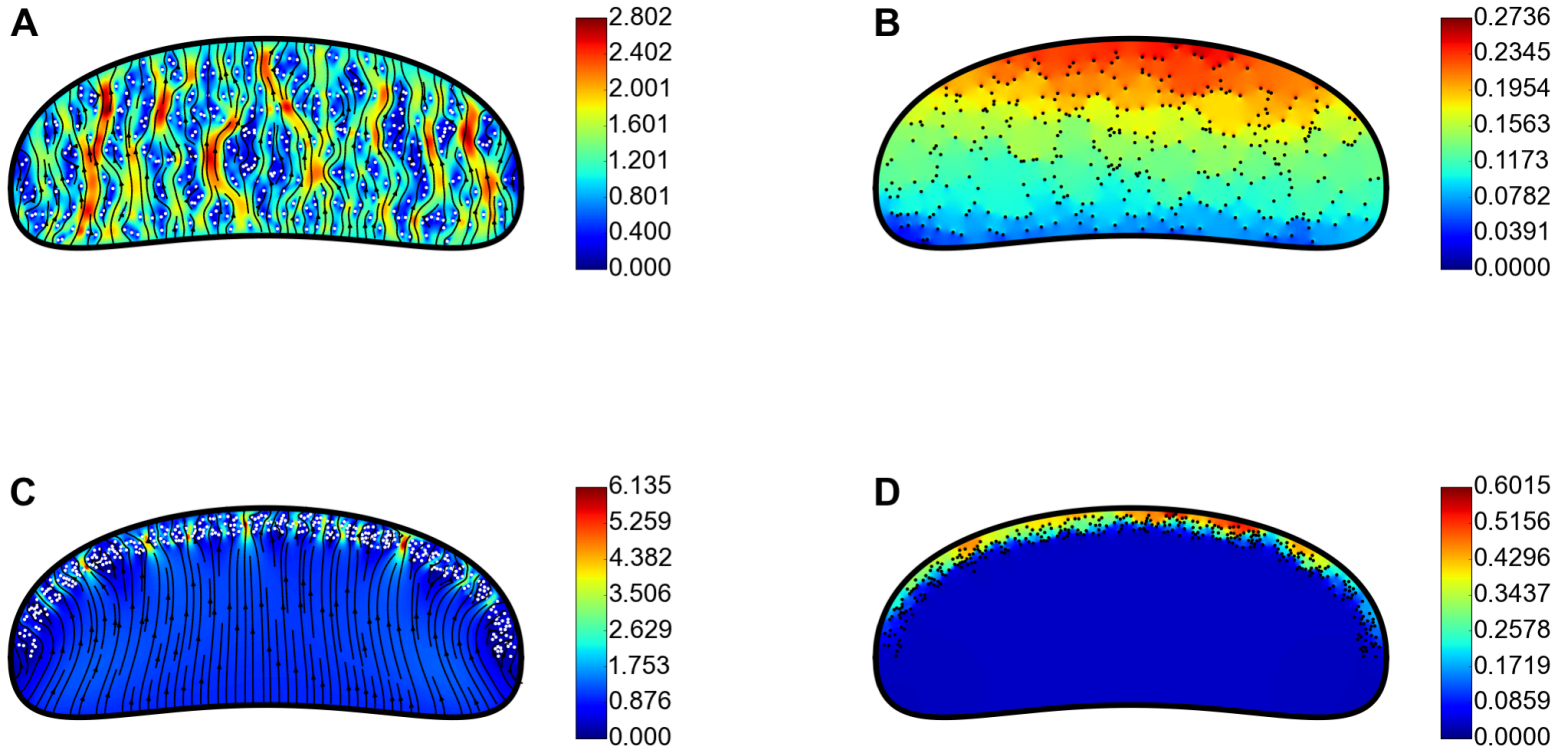
Computed membrane velocity and tension. A–B: Computed membrane velocity (A and C, units of cell speed) and tension (B and D, units of pN/µm) for 350 uniformly distributed (A,B) and front loaded (C,D) proteins. Proteins are shown in white in A,C and black in B,D.

We performed numerical simulations on flat membrane domains, which can represent either ventral or dorsal membrane surfaces. For both uniform and front-loaded protein distributions, we performed simulations with total numbers of proteins ranging from 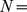
0 to 350. Fig 3 shows representative simulation results for both uniform and front-loaded distributions. Figs 3A,C and B,D show maps of magnitude and directions of flow and tension distributions, respectively, in sample simulations. More results at other protein densities and distributions are shown in Figures S1–S6. Interestingly, the flow concentrates in narrow meandering effective channels on the membrane surface. In these channels, which are effectively paths devoid of the attached proteins, the flow rate could be a few-fold higher than the cell speed.

For each simulation, we calculated the average tension drop across the cell as described above. We performed 10 simulations at each number of proteins, and computed the average front to rear tension drop over all runs at each protein number. The membrane tension increases almost linearly from the rear to the front for the random uniform distribution of the attached transmembrane proteins. When the proteins are front-loaded, the same proteins number as in the uniform case produces a greater total average tension increase from the rear to the front. This suggests that as the proteins become more tightly packed, they restrict the flow more than an equal number of well-spaced proteins, as expected.

### 3. Dependence of the membrane tension gradient on transmembrane protein size and area fraction in the 2D model

No matter how much resistance the transmembrane proteins generate, the average membrane flow rate in the 2D model is of the order of the cell speed. It is the membrane tension gradient that must adjust to maintain this flow rate. The Saffman-Delbruck formula (2) allows us to estimate the drag due to one transmembrane protein. For the parameter values given in Table 1, the drag coefficient is 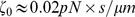. In the linear approximation, the drag imposed by 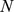 proteins is additive, and the rear-to-front difference in membrane tension can be estimated as: 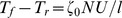
(14)where 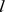 is the lateral width of the lamellipodium.

**Figure 4:**
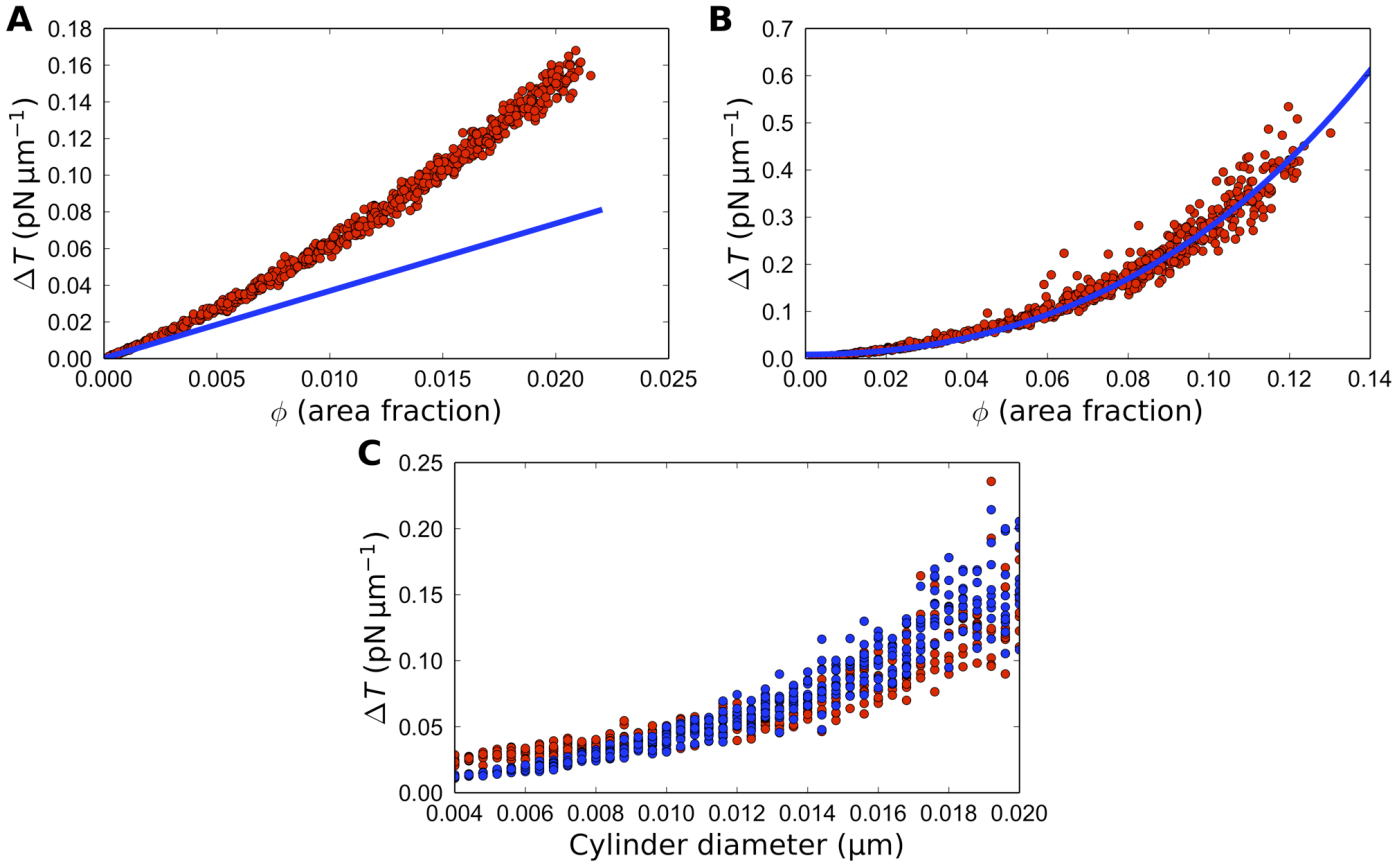
Computed membrane tension gradient. A: Computed average membrane tension drop as a function of the area fraction of proteins for uniform distributions of proteins (red) compared with the analytic linear prediction (blue). B: Computed average membrane tension drop as a function of the area fraction of proteins for front-loaded proteins (red) compared with the analytic linear prediction from equation (16) (blue). The data in (A–B) are for variable number of proteins 0.1 µm in diameter. C: Numerical results (red) and equation (16) fit (blue) for tension drop as a function of protein diameter. The data points include front loaded simulations and show individual simulation results from the runs with varied diameters and protein number equal to 100. Other parameters are listed in Table 1.

The computations of the 2D flow allow us to investigate how the rear-to-front difference in membrane tension depends on protein density. We plotted respective results in Fig 4A,B. At low protein densities, there is good agreement between the analytic prediction (14) and our numerical results (Fig 4A). Our numerical simulations show that this linear approximation, however, is valid only up to the density of about one ‘buoy’ per square micron (or area fraction *φ* occupied by proteins less than 1%), in which case 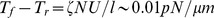, which is very low compared to the measurements in the neuronal growth cone and which is well below the experimental noise in the tether-pulling experiments.

At higher protein densities, however, both the uniform and front-loaded protein distributions quickly produce a much larger tension drop than would be predicted by the linear theory because closely spaced proteins affect the flow in a non-additive way, which is a well-known hydrodynamic phenomenon. Indeed, there is a certain analogy between the 2D membrane flow through a ‘maze’ of disks and 3D groundwater flow through a maze of spheroid objects. In the latter case, the well-known Kozeny-Carman equation describes the pressure drop 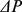 for a fluid of viscosity 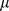 flowing a distance 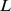 through a porous medium at an average velocity 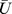 [42]: 
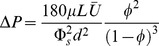
(15)


The porous medium is assumed to be made up of randomly placed, immovable particles that can be described by their sphericity 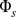, which is a measure of how spherical the particles are (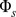 = 1 for spheres), the diameter 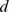 of a sphere having the same volume as one of the particles, and the volume fraction *φ*, which is the fraction of total volume that is occupied by the solid particles.

We fitted our numerical data to a similar equation of the form: 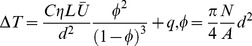
(16)


Here 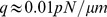 is a small correction that stems from the fact that there is a small viscous resistance to the membrane flow even in the absence of the protein obstacles, 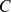 is the dimensionless parameter to be found from fitting, and 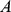 is the area on which 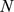 disc-like obstacles are randomly placed. We minimized the least squared error over all our simulation runs, and found that for 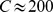 equation (16) is an excellent fit for our simulations (Fig 4B). Note that for values of the area fraction *φ* below a few percent, the numerical data shows agreement with the linear tension-density relation, not with the Kozeny-Carman quadratic relation. The reason is that the Kozeny-Carman relation comes partially from considering flow through narrow, twisting channels, and the geometry of the flow becomes quite different at very low protein density. However, tension gradients of biological interest develop at higher protein density, so for practical purposes equation (16) is valid. To additionally test equation (16) we did computations with 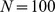 proteins varying the protein diameter. Note that when parameter 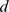 varies, so does parameter *φ*, so we plotted the results as shown in Fig 4C. The fit generated by equation (16) to this numerical data is good.

It is unknown what fraction of the transmembrane proteins covering a few tens of per cent of the plasma membrane area is attached to the cytoskeleton. It would be reasonable to assume that about 10% of the whole membrane area is covered by such attached proteins. Assuming *φ* = 0.1, we use equation (16) to estimate the tension gradient for 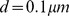 (respective protein density is 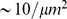): 
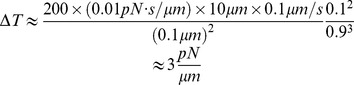
.

This estimate is very similar to the measurement in the neuronal growth cones [17].

So far we reported the simulations using the value of 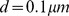 characteristic for nascent adhesion protein complexes at the leading edge [43]. Single proteins are smaller; the characteristic size is 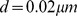 [44]. With such a value of this parameter, the estimate for the tension gradient for *φ* = 0.1 becomes 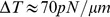, which is a great tension gradient, comparable in fact to the average membrane tension in many cells. (Respective protein density is 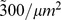). Here we report two simulations with the parameter value 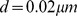 that we performed for front-loaded proteins to verify that the predictions made from our simulations with 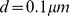 are consistent with simulations at a smaller protein diameter. Figures S7 and S8 show individual simulations with 2000 and 3000 transmembrane proteins of diameter 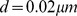, respectively. These two simulations had area fractions of *φ* = 0.023 and 0.036, respectively. These simulations gave membrane tension drops of 1.03 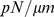 and 2.65 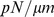, respectively, on the same order as the values 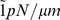 predicted by our analytical fit. Further work is necessary to determine whether our analytic fit is as good in this small diameter regime as it is for larger diameters.

Since the membrane is a very viscous fluid, the tension and velocity distributions in the plane of the membrane reach steady state for a given transmembrane protein distribution almost instantaneously [1], so we can predict the tension and flow for a given transmembrane protein distribution without regard to the timescale over which these adhesions move relative to the cell boundary. However, to verify that our steady-state computations of membrane tension were valid over the timescales relevant for cell motility, we simulated an individual membrane with 100 embedded transmembrane proteins for 200 seconds. As in our other simulations, we computed the membrane flow velocity and tension in the lab frame, but we allowed the membrane boundary to move forward relative to the adhesions at the speed of the cell. To remain consistent with our simulations of transmembrane proteins concentrated near the leading edge, as the cell moved forward we removed any transmembrane proteins that became more than 1 µm from the leading edge, and replaced those proteins with new, randomly generated ones within 1 µm of the leading edge. Movies S1 and S2 (online) show the results of this simulation for both flow and tension. In the movies, all results are given in the lab frame, but we show a field of view that keeps pace with the boundary of the membrane. Figure S9 shows in red the spatial average membrane tension drop over the cell as a function of time, and in blue shows the predicted tension drop from the analytical fit for this cell, computed from the average value of *φ* for this cell over the 200 seconds of simulated time.

### 4. Tank-tread flow versus no membrane flow in the framework of the moving cell

Examination of the simple 1D model of membrane flow in the side view of the cell raises an interesting issue that stems from the fact that in most motile cells, there is a very high density of attachments of adhesion molecular complexes that go through the ventral membrane to the actin network, while at the dorsal membrane, the molecular nature and number of the actin-membrane attachments is likely very different. This suggests that the membrane flows differently at the ventral and dorsal surfaces, as the effective viscous drag coefficients are scaled by protein densities. Indeed, the analytical solution for the three linear equations (8–9), 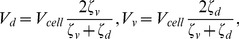
(17)implies that if there is the same density of transmembrane proteins attached to the cytoskeleton on the dorsal and ventral surfaces (so that 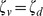) then the dorsal and ventral flows would be the same in the lab coordinate system (meaning no flow in the cell frame). It would be very surprising if there is the same density of transmembrane proteins attached to the cytoskeleton on the dorsal and ventral surfaces and so in general for 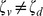, the model predicts a tank-tread flow. How can we reconcile this prediction with the fact that in a number of cell types no flow in the cell frame is observed?

There are reports on abnormally low diffusion coefficients of membrane-associated proteins at the very leading edge of motile cells [39], [40], suggesting that the leading edges harbors especially high concentrations of proteins, many of which could be linked to the cytoskeleton. If this is the case, then the tank-tread membrane flow across the leading edge would be largely obstructed by this ‘protein crust’, and conservation of lipid number would require that both dorsal and ventral flow in this case be equal to the cell speed in the lab coordinate system: 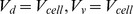. Interestingly, this means that the tensions at the leading edge at ventral and dorsal membrane surfaces are different (Fig 1E): 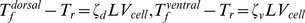
(18)


An experiment testing this prediction – pulling membrane tethers from ventral and dorsal leading edge and measuring respective forces – would be very difficult, but not impossible.

### 5. Influence of membrane-cytoskeleton attachments on cell locomotion

To investigate the effects of intracellular membrane transport and force balances at the front and rear of the motile cell on the membrane flow and tension gradient, we solve four linear equations (10–13). The solution has the form: 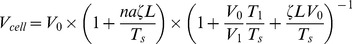
(19)


This formula shows that the cell speed is accelerated by intracellular membrane transport and is slowed down by the mechanical resistance of the rear (the first term in the second bracket) and of the front (the second term in the second bracket). The influence of intracellular membrane transport is determined by the relative magnitude of the effective speed of this transport, 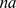, and of the membrane flow speed generated by the stall tension (membrane tension that stalls the actin protrusion) against the transmembrane viscous resistance, 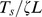.

The overall rates of transport between internal membranes and the plasma membrane vary among different cell types. In Dictyostelium amoeba it takes a few minutes to replace an area equal to the cell surface area [45] whereas in fibroblasts it takes approximately one hour [46]. If it takes 500 s to replace the whole plasma membrane, while it takes about 100 s to move one body length, then equation (11) suggests that 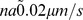. From [41], 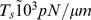, and our estimate of the transmembrane viscous resistance above is 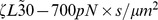. Then, 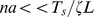 and so the mechanical influence of intracellular membrane transport on the cell speed is minuscule. However, if the stall tension is an order of magnitude lower in non-keratocyte cells, while the membrane resistance is higher, then it is possible that intracellular membrane transport becomes the limiting factor in cell locomotion.

Based on the analysis of the keratocyte fragments in [10], the ratio of the stall force to the free polymerization rate 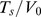 is of the same order of magnitude as the ratio of the characteristic tension and motion rate at the rear 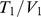 , so the factor 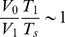. Thus, the influence of the membrane flow on the cell speed is determined by the ratio of the free polymerization rate 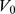 to the membrane flow speed generated by the stall tension against the transmembrane viscous resistance, 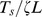. The free polymerization rate could be a few-fold greater than the observed cell speed. Taking 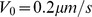, we estimate the ratio 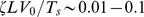, depending on the value of 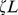. Thus, the effect of the transmembrane proteins' viscous drag is small but could be noticeable in slowing down cell movement. Note that in the limit of extremely high viscous drag 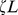, 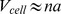, tension at the front is almost at stall, and tension at the rear is very small.

Membrane tension at the front can also be easily estimated from equations (10–13): 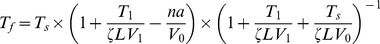
(20)


This tension is relieved by intracellular membrane transport (negative term in the first bracket), but this effect is quite small for keratocytes because according to our estimates 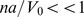. This transport, however, can significantly relieve tension for slow-moving cells. The greater the transmembrane proteins' viscous resistance 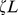, the greater the membrane tension at the front, and in the limit of high viscous resistance the membrane tension approaches the stall tension. Our estimates show that if 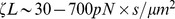, then the transmembrane proteins' viscous drag increases the membrane tension at the leading edge by at most ∼10%. The approximate value of the leading edge tension in this case is 
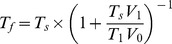
, and at the rear the tension is almost the same.

## Discussion

In this paper, we estimated the gradient in membrane tension from the rear to the front of the cell necessary to maintain the membrane flow that keeps up with cell locomotion. This gradient is predicted to be of the order of a few pN/µm if the area fraction of the transmembrane protein complexes attached to actin is about 10% and if their diameter is of the order of 0.1 µm. However, if at the same area fraction the protein diameter is of the order of 20 nm, then the tension gradient becomes tens of pN/µm. The apparent average membrane tension extracted from tether-force measurement values ranges from a few pN/µm in neuronal growth cones [21] to hundreds of pN/µm in keratocytes [22]. Thus, the tension gradient could be comparable to the average membrane tension and detectable in principle if membrane tethers are pulled from the front and rear of the cell. The caveat to interpretation of such measurements though is that it is not easy to separate the in-plane membrane tension from the effective tension due to membrane-cytoskeleton attachment. Another interesting model prediction is that the membrane flow could concentrate in narrow meandering effective channels on the membrane surface.

Furthermore, this tension gradient is generated by the actin network pushing the leading edge forward, and this force is of the order of hundreds of pN/µm [22], [41]. Thus, in principle a non-negligible part of this pushing force has to support membrane transport, in addition to pulling up the cell rear and overcoming resistance of the extracellular environment. We estimate that if the area fraction of the actin-attached transmembrane proteins becomes higher than 10%, the force needed to generate the membrane flow becomes non-negligible, and at 20% it becomes rate-limiting. With such a high area fraction, the directed intracellular membrane traffic can also become the principal mechanism of membrane recycling.

Our model predicts that in general, if the plasma membrane is an interconnected fluid domain then a tank-tread mode of the membrane flow has to be observed. The fact that in the majority of cases where the membrane flow was measured the flow does not show such a pattern [14]–[18] may indicate that at the leading edge the membrane is not fluid but rather a non-flowing (or very slowly flowing) ‘protein crust’ interspersed with lipids trapped in protein ‘corrals’. We predict that in this case the membrane tensions at the ventral and dorsal leading edges are different.

Our model has a number of limitations that stem from simplifying assumptions that we made in order to increase its transparency and tractability. We considered a constant rate of intracellular membrane traffic from the rear to the front, implicitly assuming that the rates of exocytosis at the front and endocytosis at the rear are not rate limiting. This issue has to be researched further. In addition, measurements have revealed that rates of endocytosis decrease as membrane tension increases [47], and exocytosis is likely to also be tension-dependent [20]. In the future, the model can be extended to include a more detailed and realistic sub-model for intracellular membrane recycling. We also did not explicitly consider a possible contribution to the membrane tension from adhesion breaking at the very rear of the cell, however, mathematically, this contribution can be included in model parameters 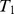 and 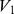.

The shear between the substrate and ventral membrane for the motile cell is negligible. Respective mechanical contributions can be estimated as water viscosity times cell speed divided by the characteristic distance 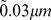 between the ventral surface and the substrate and multiplied by the cell length: 
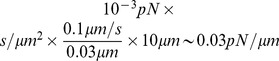
. On the other hand, we likely underestimate the membrane tension gradient because we did not consider the effect of hydrodynamic interactions with multiple actin filaments, some of which are proximal to the plasma membrane. This effect was recently investigated [24], and it is not negligible. The authors of [24] computed analytically the membrane flow and forces generated by a number of proteins anchoring the actin network to the dorsal and ventral membrane sandwiched between two solid surfaces and found that the effective friction between the proteins anchoring the actin network to the membrane and the surface can propel a cell in the absence of any direct adhesion mechanism with velocities comparable to velocities of adhesive cells. The geometry of the problem, methods and goals of that important study are different from those in our paper, yet [24] also demonstrates that the membrane flow and associated forces could be important in the cell motility process.

In addition, effects of inhomogeneous distributions of many types of lipids and proteins in the plane of the plasma membrane and of various diffusion coefficients of the lipids and proteins on the flow and tension gradient in many cells also remain unclear. Moreover, most cells migrate in a non-steady way, so time dependent non-steady solutions for the membrane flow equations have to be explored to investigate membrane recycling in these cells. Also, there is often a significant retrograde flow of the actin network near the leading edge of motile cells. Many transmembrane proteins at the dorsal surface attached to actin should then flow to the rear of the cell, while some adhesions at the ventral surface remain stationary in the lab frame, and some are also drifting to the rear [29]. Our model can be easily extended to simulate this situation, but qualitatively it is clear that the effect of the actin retrograde flow will lead to further increase of the resistance to the forward membrane flow and of the tension gradient.

Last, but not least, perhaps the most interesting open problem stems from the fact that in most cells the plasma membrane enveloping the cell is not pulled taut, but rather is folded, ruffled and invaginated [48] in multiple local membrane reservoirs, i.e. cup-shaped caveolae [49]. These folds enable motile cells to undergo fast changes in cell surface area [48], and it is likely that a significant effect of the membrane fold dynamics on the forward membrane flow and tension gradient will be to ease the flow and decrease the necessary membrane tension gradient. Future modeling will clarify how important this effect is.

## Methods

The numerical method used to produce the solutions to Stokes equations is the method of regularized Stokeslets. This method was introduced by Cortez, and the details of it are given in [50]. The method uses a boundary integral approach to approximate the solution to Stokes equations driven by a discrete collection of prescribed point forces. The velocity and pressure at any point can be computed by summing the contributions to the solution from each force. Alternatively, the method can be used to find the solution to Stokes equations for a set of points with prescribed velocities. For this second approach, a 2N×2N linear system (where N is the number of points) is solved to compute the force at each point. Those forces can then be used to compute the solution at any point in the plane. We used this second approach to prescribe the velocity of the membrane at the boundary of the membrane and at the boundary of each transmembrane protein.

### Discretizing the cell

In order to use the method of regularized Stokeslets to compute membrane flow, we first needed to discretize both the cell boundary and the boundaries of the transmembrane proteins into a collection of points. To determine the spacing between neighboring points in our discretization, we started by discretizing the transmembrane proteins. For most of our simulations, we used circular proteins with a diameter of *d* = 0.1 µm. At this radius, the numerical method worked well with 25 equally spaced proteins around the boundary of the circle. This gave a spacing of *s*  =  π*d* = 25 = 0.0126 µm between neighboring points. We used this fixed spacing for all our simulations, including on the cell boundary and on circular proteins of varying radii. As suggested in [50], we use a blob function *φ_ε_* with the spreading parameter ε chosen to scale with this spacing *s*. We found that simply using ε = *s* worked well. Discretizing the cell boundary required us to modify the shape of the cell. The original cell shape is given by equations (4,5). To generate equally spaced collections of points along each of these curves, it is necessary to compute each curve's arc length, which requires numerical evaluation of the integral 
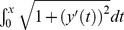
. The derivatives of (4,5) are both singular near *x* = 0 and *x* = 1, which made this arc length computation highly unstable. To fix this, we modified both curves, defining the shape of the cell as: 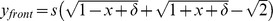, 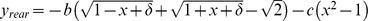 for a small value of the parameter δ. In our simulations, we took δ = 0.00001, which was enough for us to compute the arc length without difficulty.

### Computing tension drops

With the cell boundary and proteins discretized, it is easy to use the method of regularized Stokeslet. We simply prescribed velocities at each of our boundary points according to the

GRE model, and prescribed a zero velocity at each point on a protein boundary. Inverting the mobility matrix as described in [50] gives us the appropriate force at each boundary point, from which we can compute pressure and membrane flow everywhere within the cell. Our main use of this technique was to integrate pressure at the leading and rear edges of the cell to determine average lead and rear pressures, from which we computed the tension drop over the length of the cell. The only complication to this approach was that the numerical method causes a sharp jump in the pressure very near the cell boundaries, as shown in Figure S10. This jump causes evaluations of the pressure on the boundary itself to be inaccurate. To rectify the inaccuracies caused by the jump at the boundary, we simply used pressure values that were very close to, rather than on, the leading edge. To integrate the pressure at the leading edge, we instead integrated over a curve parallel to the leading edge, but offset a distance of 4*s* = 0.0503 µm towards the interior of the cell, and similarly for the trailing edge. As can be seen in Figure S10, computing pressure values offset by this small amount was sufficient to avoid error caused by the jumps at the boundaries.

## Supporting Information

Figure S1 **Computed membrane flow in cell without transmembrane proteins.** Black lines and arrows give streamlines of the membrane velocity, while the color plot gives the local speed. The speed in this and all next supplementary figures is reported in units of the steady cell speed.
(PDF)

Figure S2 **Computed membrane velocity (top) and tension (bottom) for 30 uniformly and randomly distributed proteins of diameter 0.1 µm.** Proteins are shown in white in the top figure and black in the bottom figure. The speed in this and all other figures is reported in units of µm/s. The tension in this and all next supplementary figures is reported in units of pN/µm.
(PDF)

Figure S3 **Computed membrane velocity (top) and tension (bottom) for 30 front-loaded proteins of diameter 0.1 µm.** Proteins are shown in white in the top figure and black in the bottom figure.
(PDF)

Figure S4 **Computed membrane velocity (top) and tension (bottom) for 100 front-loaded proteins of diameter 0.04 µm.** Proteins are shown in white in the top figure and black in the bottom figure.
(PDF)

Figure S5 **Computed membrane velocity (top) and tension (bottom) for 100 front-loaded proteins of diameter 0.1 µm.** Proteins are shown in white in the top figure and black in the bottom figure.
(PDF)

Figure S6 **Computed membrane velocity (top) and tension (bottom) for 100 front-loaded proteins of diameter 0.2 µm.** Proteins are shown in white in the top figure and black in the bottom figure.
(PDF)

Figure S7 **Computed membrane velocity (top) and tension (bottom) for 2000 front-loaded proteins of diameter 0.02 µm.**
(PDF)

Figure S8 **Computed membrane velocity (top) and tension (bottom) for 3000 front-loaded proteins of diameter 0.02 µm.**
(PDF)

Figure S9 **The membrane tension drop as a function of time.** The membrane tension drop over the cell as a function of time (red), and the predicted tension drop from the analytical fit for this cell (blue), computed from the average values in the simulation of the moving cell over 200 seconds of simulated time. About 100 front-loaded proteins of diameter 0.1 µm are stationary, appear at the leading edge and disappear at the rear edge of the protein-loaded band, while the cell moves forward.
(PDF)

Figure S10 **Pressure near cell boundaries.** A: Pressure (pN/µm) in a simulated cell, with blue line through middle of the cell. B: Cross section plot showing pressure in cell along the cell's midline. Note that the pressure jumps sharply near both the rear and leading edges. C–D: Close up of the pressure cross section near the rear (C) and front (D) of the cell. Note the sharp jumps in pressure over very small spatial scales. In red (dots and dashed line), we show the position 4s≈0.05 µm away from the edges used for actual computation of edge pressures. Note also that membrane tension is equivalent to the pressure with the minus sign.
(PDF)

Movie S1 **Membrane flow speed distribution in steadily moving cell.** Results of the simulations with about 100 embedded transmembrane proteins for 200 seconds. As in other simulations, the membrane flow velocity and tension are computed in the lab frame, but the membrane boundary is allowed to move forward at the speed of the cell. As the cell moves forward, any transmembrane proteins that are displaced more than 1 micron from the leading edge are removed and replaced with new, randomly generated ones within 1 micron of the leading edge. The results are shown in a field of view that keeps pace with the boundary of the membrane.
(MP4)

Movie S2 **Membrane tension distribution in steadily moving cell.** This movie shows the results for the tension of the simulations described in the legend of the Movie S1.
(MP4)

## References

[1] Keren K (2011) Cell motility: the integrating role of the plasma membrane. Eur Biophys J 40: 1013–1027. 21833780 10.1007/s00249-011-0741-0 PMC3158336

[2] Bretscher MS, Aguado-Velasco C (1998) Membrane traffic during cell locomotion. Curr Opin Cell Biol 10: 537–41. 9719876 10.1016/s0955-0674(98)80070-7

[3] Houk AR, Jilkine A, Mejean CO, Boltyanskiy R, Dufresne ER, et al (2012) Membrane tension maintains cell polarity by confining signals to the leading edge during neutrophil migration. Cell 148: 175–188. 22265410 10.1016/j.cell.2011.10.050 PMC3308728

[4] Takano K, Toyooka K, Suetsugu S (2008) EFC/F-BAR proteins and the N-WASP–WIP complex induce membrane curvature-dependent actin polymerization. EMBO J 27: 2817–2828. 18923421 10.1038/emboj.2008.216 PMC2580791

[5] Bray D (2000) Cell Movements: From Molecules to Motility. New York: Garland Science. 392 p.

[6] Pollard TD, Borisy GG (2003) Cellular motility driven by assembly and disassembly of actin filaments. Cell 112: 453–465. 12600310 10.1016/s0092-8674(03)00120-x

[7] Ridley AJ, Schwartz MA, Burridge K, Firtel RA, Ginsberg MH, et al (2003) Cell migration: integrating signals from front to back. Science 302: 1704–1709. 14657486 10.1126/science.1092053

[8] Anderson KI, Wang YL, Small JV (1996) Coordination of protrusion and translocation of the keratocyte involves rolling of the cell body. J Cell Biol 134: 1209–1218. 8794862 10.1083/jcb.134.5.1209 PMC2120980

[9] Gomes ER, Jani S, Gundersen GG (2005) Nuclear movement regulated by Cdc42, MRCK, myosin, and actin flow establishes MTOC polarization in migrating cells. Cell 121: 451–463. 15882626 10.1016/j.cell.2005.02.022

[10] Ofer N, Mogilner A, Keren K (2011) Actin disassembly clock determines shape and speed of lamellipodial fragments. Proc Natl Acad Sci U S A 108: 20394–20399. 22159033 10.1073/pnas.1105333108 PMC3251093

[11] Fujiwara T, Ritchie K, Murakoshi H, Jacobson K, Kusumi A (2002) Phospholipids undergo hop diffusion in compartmentalized cell membrane. J Cell Biol 157: 1071–1081. 12058021 10.1083/jcb.200202050 PMC2174039

[12] Fletcher SJ, Rappoport JZ (2010) Moving forward: polarized trafficking in cell migration. Trends Cell Biol 20: 71–78. 20061150 10.1016/j.tcb.2009.11.006

[13] Keren K, Pincus Z, Allen GM, Barnhart EL, Marriott G, et al (2008) Mechanism of shape determination in motile cells. Nature 453: 475–480. 18497816 10.1038/nature06952 PMC2877812

[14] Kucik DF, Elson EL, Sheetz MP (1989) Forward transport of glycoproteins on leading lamellipodia in locomoting cells. Nature 340: 315–317. 2473406 10.1038/340315a0

[15] Kucik DF, Elson EL, Sheetz MP (1990) Cell migration produces membrane flow. J Cell Biol 111: 1617–1622. 2211827 10.1083/jcb.111.4.1617 PMC2116247

[16] Lee J, Ishihara A, Theriot JA, Jacobson K (1993) Principles of locomotion for simple-shaped cells. Nature 362: 167–171. 8450887 10.1038/362167a0

[17] Lee J, Gustafsson M, Magnusson KE, Jacobson K (1990) The direction of membrane lipid flow in locomoting polymorphonuclear leukocytes. Science 247: 1229–1233. 2315695 10.1126/science.2315695

[18] Traynor D, Kay RR (2007) Possible roles of the endocytic cycle in cell motility. J Cell Sci 120: 2318–2327. 17606987 10.1242/jcs.007732

[19] Dai J, Sheetz MP (1995) Axon membrane flows from the growth cone to the cell body. Cell 83: 693–701. 8521486 10.1016/0092-8674(95)90182-5

[20] Sheetz MP, Dai J (1996) Modulation of membrane dynamics and cell motility by membrane tension. Trends Cell Biol 6: 85–89. 15157483 10.1016/0962-8924(96)80993-7

[21] Dai J, Sheetz MP (1999) Membrane tether formation from blebbing cells. Biophys J 77: 3363–3370. 10585959 10.1016/S0006-3495(99)77168-7 PMC1300608

[22] Lieber AD, Yehudai-Resheff S, Barnhart EL, Theriot JA, Keren K (2013) Membrane tension in rapidly moving cells is determined by cytoskeletal forces. Cur Biol 23: 1409–1417. 10.1016/j.cub.2013.05.063 23831292

[23] Evans EA, Skalak R (1979) Mechanics and thermodynamics of biomembranes: part 1. CRC Crit Rev Bioeng 1979 3: 181–330. 393460

[24] Schweitzer J, Kozlov MM (2013) Cell motion mediated by friction forces: understanding the major principles. Soft Matter 9: 5186–5195.

[25] Cooper GM (2000) The cell: A molecular approach. Sunderland: Sinauer Associates. 832 p.

[26] Diz-Munoz A, Krieg M, Bergert M, Ibarlucea-Benitez I, Muller DJ, et al (2010) Control of directed cell migration in vivo by membrane-to-cortex attachment. PLoS Biol 8: e1000544. 21151339 10.1371/journal.pbio.1000544 PMC2994655

[27] Sheetz MP, Sable JE, Döbereiner HG (2006) Continuous membrane-cytoskeleton adhesion requires continuous accommodation to lipid and cytoskeleton dynamics. Annu Rev Biophys Biomol Struct 35: 417–434. 16689643 10.1146/annurev.biophys.35.040405.102017

[28] Doherty GJ, McMahon HT (2008) Mediation, modulation, and consequences of membrane-cytoskeleton interactions. Annu Rev Biophys 37: 65–95. 18573073 10.1146/annurev.biophys.37.032807.125912

[29] Gardel ML, Sabass B, Ji L, Danuser G, Schwarz US, et al (2008) Traction stress in focal adhesions correlates biphasically with actin retrograde flow speed. J Cell Biol 183: 999–1005. 19075110 10.1083/jcb.200810060 PMC2600750

[30] Wessels D, Reynolds J, Johnson O, Voss E, Burns R, et al (2000) Clathrin plays a novel role in the regulation of cell polarity, pseudopod formation, uropod stability and motility in Dictyostelium. J Cell Sci 113: 21–36. 10591622 10.1242/jcs.113.1.21

[31] Howes MT, Kirkham M, Riches J, Cortese K, Walser PJ, et al (2010) Clathrin-independent carriers form a high capacity endocytic sorting system at the leading edge of migrating cells. J Cell Biol 190: 675–691. 20713605 10.1083/jcb.201002119 PMC2928008

[32] Saffman PG, Delbruck M (1975) Brownian motion in biological membranes. Proc Natl Acad Sci U S A 72: 3111–3113. 1059096 10.1073/pnas.72.8.3111 PMC432930

[33] Barnhart EL, Lee KC, Keren K, Mogilner A, Theriot JA (2011) An adhesion-dependent switch between mechanisms that determine motile cell shape. PLoS Biol 9: e1001059. 21559321 10.1371/journal.pbio.1001059 PMC3086868

[34] Tajparast M, Glavinovic MI (2009) Forces and stresses acting on fusion pore membrane during secretion. Biochim Biophys Acta 1788: 1009–1023. 19366587 10.1016/j.bbamem.2009.01.019

[35] Chizmadzhev YA, Kumenko DA, Kuzmin PI, Chernomordik LV, Zimmerberg J, et al (1999) Lipid flow through fusion pores connecting membranes of different tensions. Biophys J 76: 2951–2965. 10354423 10.1016/S0006-3495(99)77450-3 PMC1300267

[36] Sohn JS, Tseng YH, Li S, Voigt A, Lowengrub JS (2010) Dynamics of multicomponent vesicles in a viscous fluid. J Comput Phys 229: 119–144. 20808718 10.1016/j.jcp.2009.09.017 PMC2929801

[37] Fournier MF, Sauser R, Ambrosi D, Meister JJ, Verkhovsky AB (2010) Force transmission in migrating cells. J Cell Biol 188: 287–297. 20100912 10.1083/jcb.200906139 PMC2812525

[38] Mohl C, Kirchgessner N, Schafer C, Hoffmann B, Merkel R (2012) Quantitative mapping of averaged focal adhesion dynamics in migrating cells by shape normalization. J Cell Sci 125: 155–165. 22250204 10.1242/jcs.090746

[39] Weisswange I, Bretschneider T, Anderson KI (2005) The leading edge is a lipid diffusion barrier. J Cell Sci 118: 4375–4380. 16144867 10.1242/jcs.02551

[40] Sheetz MP, Baumrind NL, Wayne DB, Pearlman AL (1990) Concentration of membrane antigens by forward transport and trapping in neuronal growth cones. Cell 61: 231–241. 2331749 10.1016/0092-8674(90)90804-n

[41] Prass M, Jacobson K, Mogilner A, Radmacher M (2006) Direct measurement of the lamellipodial protrusive force in a migrating cell. J Cell Biol 174: 767–772. 16966418 10.1083/jcb.200601159 PMC2064331

[42] Carman PC (1937) Fluid flow through granular beds. Transactions, Institution of Chemical Engineers (London). 15: 150–166.

[43] Choi CK, Vicente-Manzanares M, Zareno J, Whitmore LA, Mogilner A, et al (2008) Actin and alpha-actinin orchestrate the assembly and maturation of nascent adhesions in a myosin II motor-independent manner. Nat Cell Biol 10: 1039–1050. 19160484 10.1038/ncb1763 PMC2827253

[44] Jiang J, Magilnick N, Tsirulnikov K, Abuladze N, Atanasov I, et al (2013) Single particle electron microscopy analysis of the bovine anion exchanger 1 reveals a flexible linker connecting the cytoplasmic and membrane domains. PLoS One 8: e55408. 23393575 10.1371/journal.pone.0055408 PMC3564912

[45] Aguado-Velasco C, Bretscher MS (1999) Circulation of the plasma membrane in Dictyostelium. Mol Biol Cell 10: 4419–4427. 10588667 10.1091/mbc.10.12.4419 PMC25767

[46] Steinman RM, Mellman IS, Muller WA, Cohn ZA (1983) Endocytosis and the recycling of plasma membrane. J Cell Biol 96: 1–27. 6298247 10.1083/jcb.96.1.1 PMC2112240

[47] Dai J, Sheetz MP (1995) Regulation of endocytosis, exocytosis, and shape by membrane tension. Cold Spring Harb Symp Quant Biol 60: 567–571. 8824429 10.1101/sqb.1995.060.01.060

[48] Gauthier NC, Fardin MA, Roca-Cusachs P, Sheetz MP (2011) Temporary increase in plasma membrane tension coordinates the activation of exocytosis and contraction during cell spreading. Proc Natl Acad Sci U S A 108: 14467–14472. 21808040 10.1073/pnas.1105845108 PMC3167546

[49] Sinha B, Koster D, Ruez R, Gonnord P, Bastiani M, et al (2011) Cells respond to mechanical stress by rapid disassembly of caveolae. Cell 144: 402–413. 21295700 10.1016/j.cell.2010.12.031 PMC3042189

[50] Cortez R (2002) The Method of regularized Stokeslets. SIAM Journal of Scientific Computing 23: 1204–1225.

[51] Waugh RE (1982) Surface viscosity measurements from large bilayer vesicle tether formation. II. Experiments. Biophys J 38: 29–37. 7074197 10.1016/S0006-3495(82)84527-X PMC1328810

