# Supplementary figures and images for: Computational Estimates of Membrane Flow and Tension Gradient in Motile Cells
Source: PLoS One. 2014 Jan 17;9(1):e84524. doi: 10.1371/journal.pone.0084524 (PMC3894945; doi:10.1371/journal.pone.0084524)

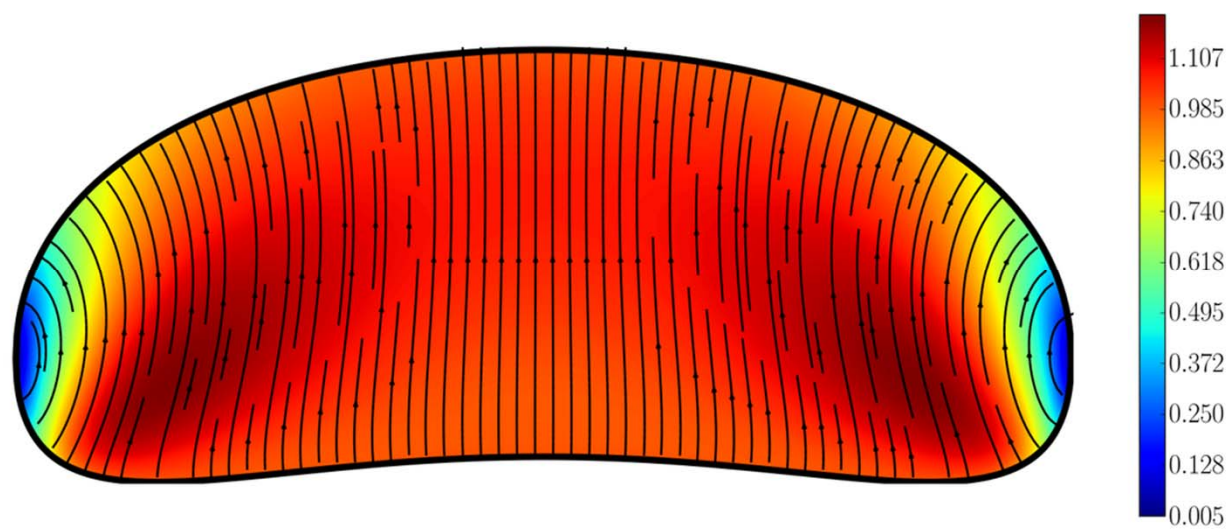

Supplement: Figure S1 — Computed membrane flow in cell without transmembrane proteins. Black lines and arrows give streamlines of the membrane velocity, while the color plot gives the local speed. The speed in this and all next supplementary figures is reported in units of the steady cell speed. (PDF) [file pone.0084524.s001.pdf]

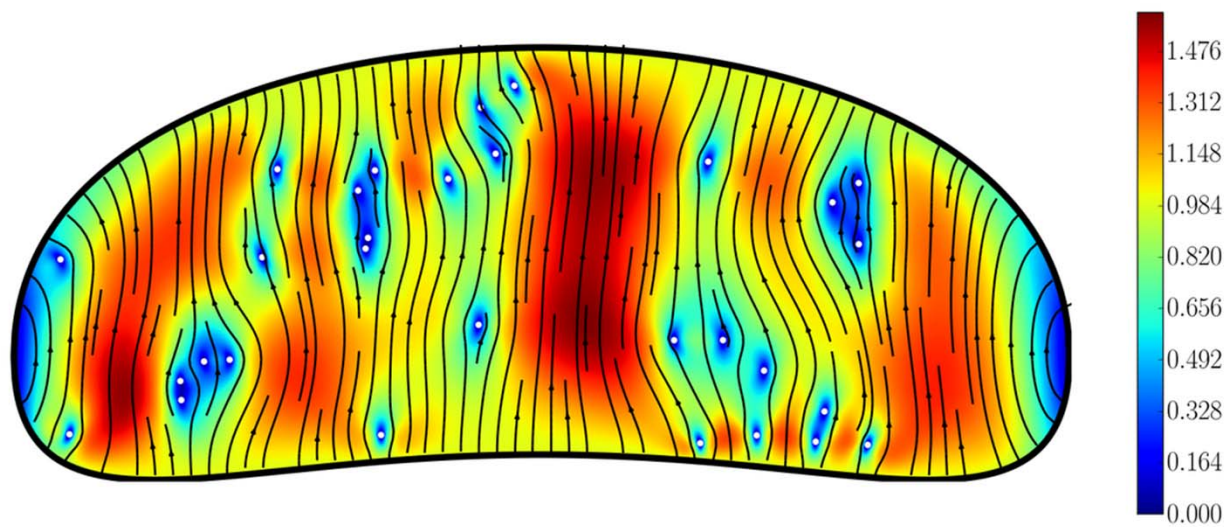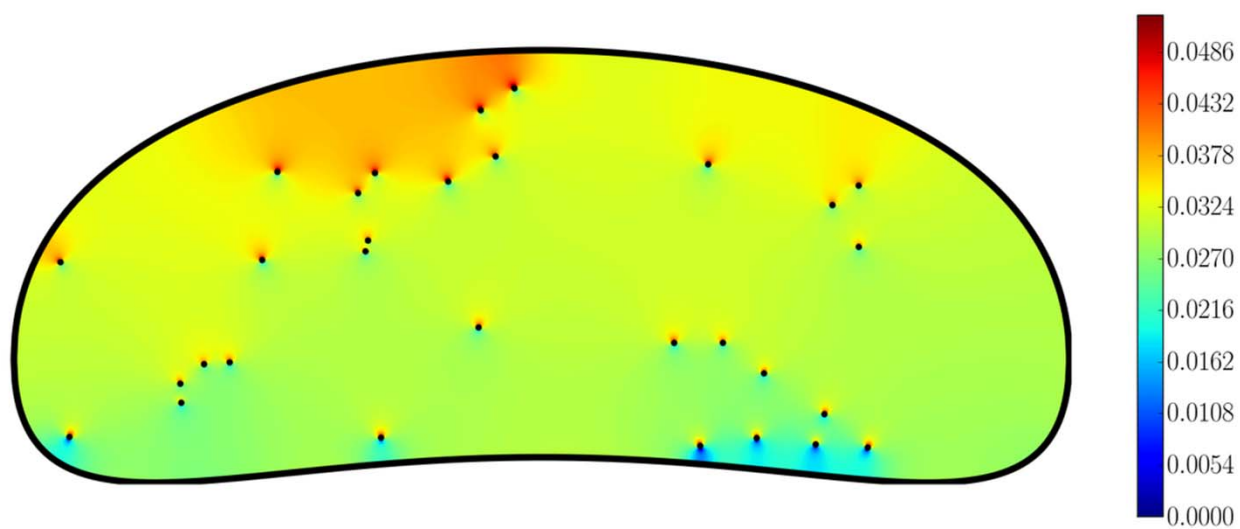

Supplement: Figure S2 — Computed membrane velocity (top) and tension (bottom) for 30 uniformly and randomly distributed proteins of diameter 0.1 µm. Proteins are shown in white in the top figure and black in the bottom figure. The speed in this and all other figures is reported in units of µm/s. The tension in this and all next supplementary figures is reported in units of pN/µm. (PDF) [file pone.0084524.s002.pdf]

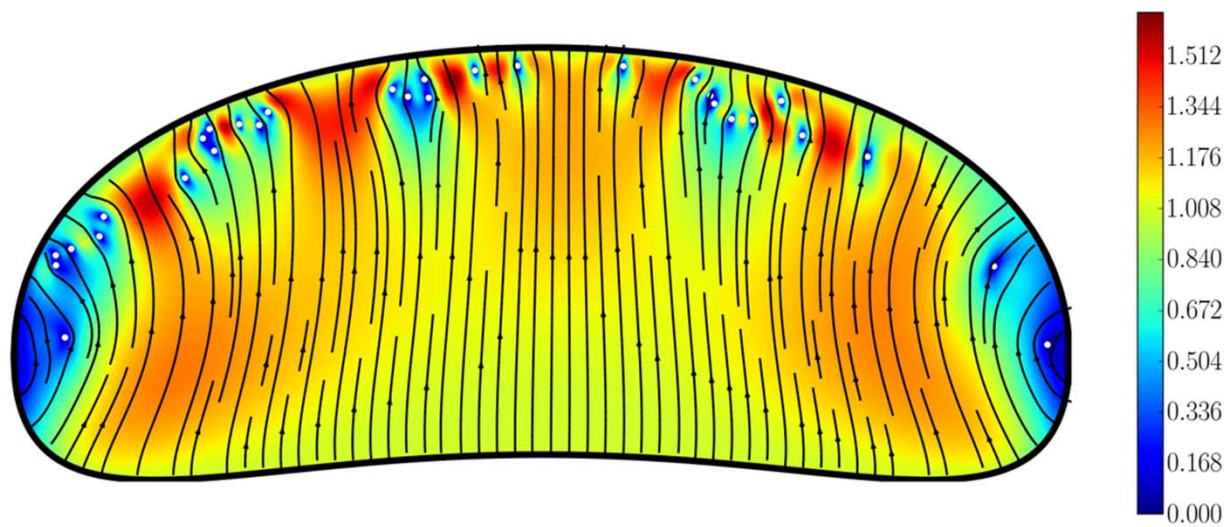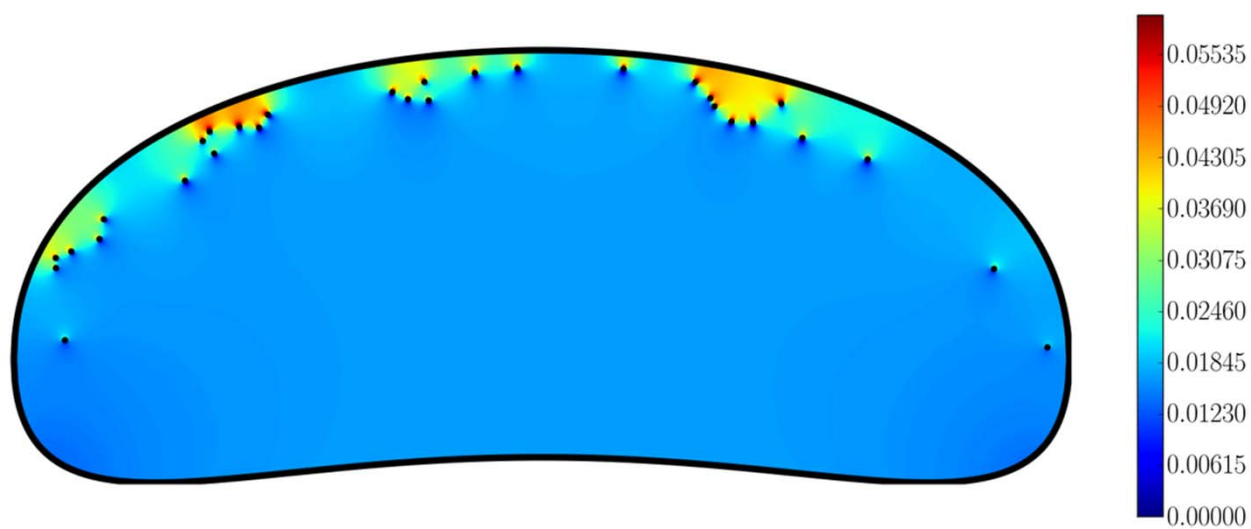

Supplement: Figure S3 — Computed membrane velocity (top) and tension (bottom) for 30 front-loaded proteins of diameter 0.1 µm. Proteins are shown in white in the top figure and black in the bottom figure. (PDF) [file pone.0084524.s003.pdf]

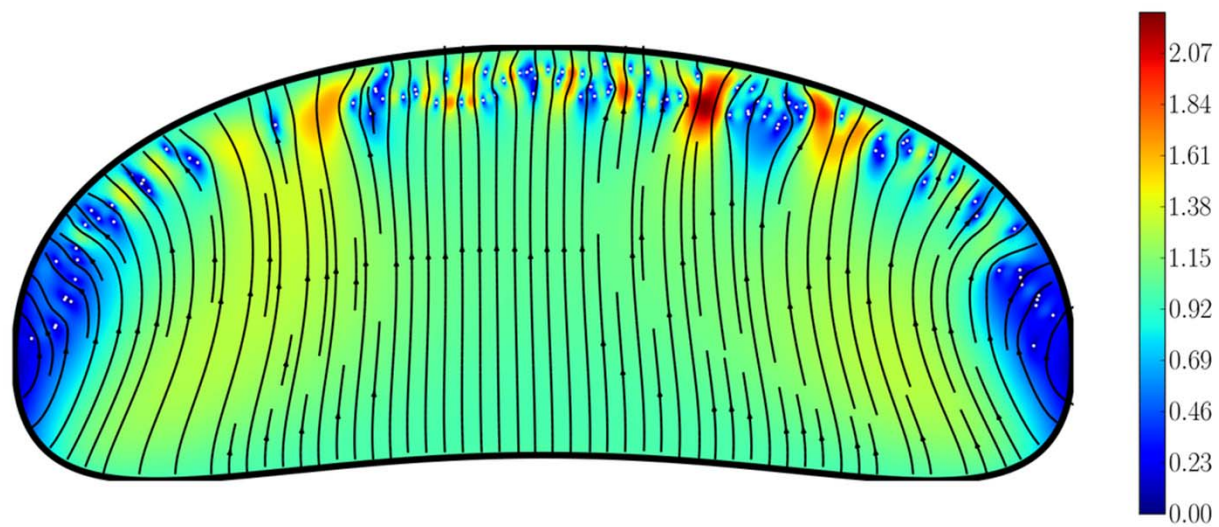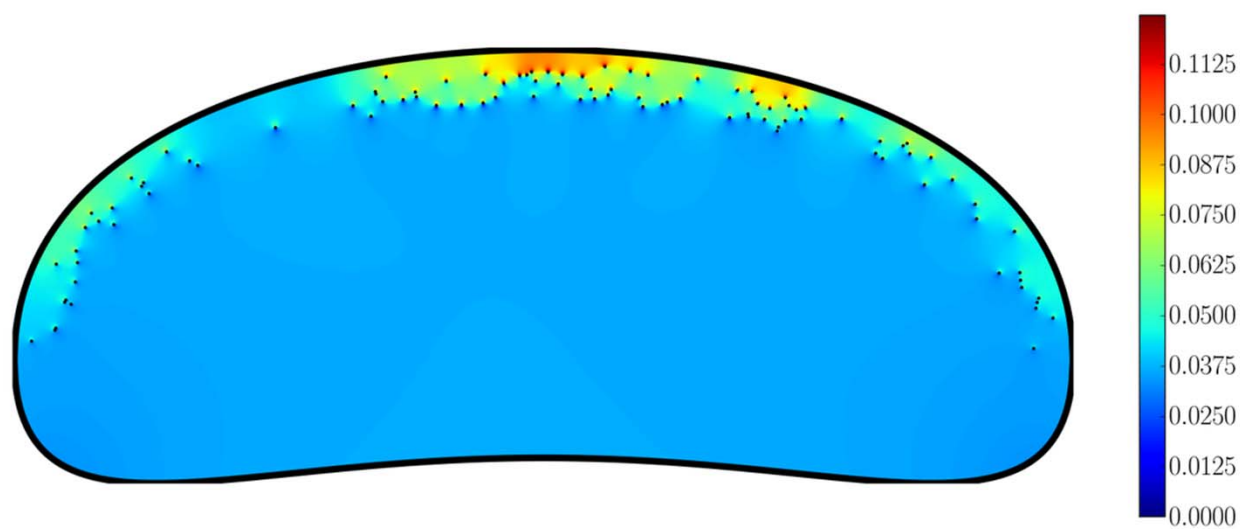

Supplement: Figure S4 — Computed membrane velocity (top) and tension (bottom) for 100 front-loaded proteins of diameter 0.04 µm. Proteins are shown in white in the top figure and black in the bottom figure. (PDF) [file pone.0084524.s004.pdf]

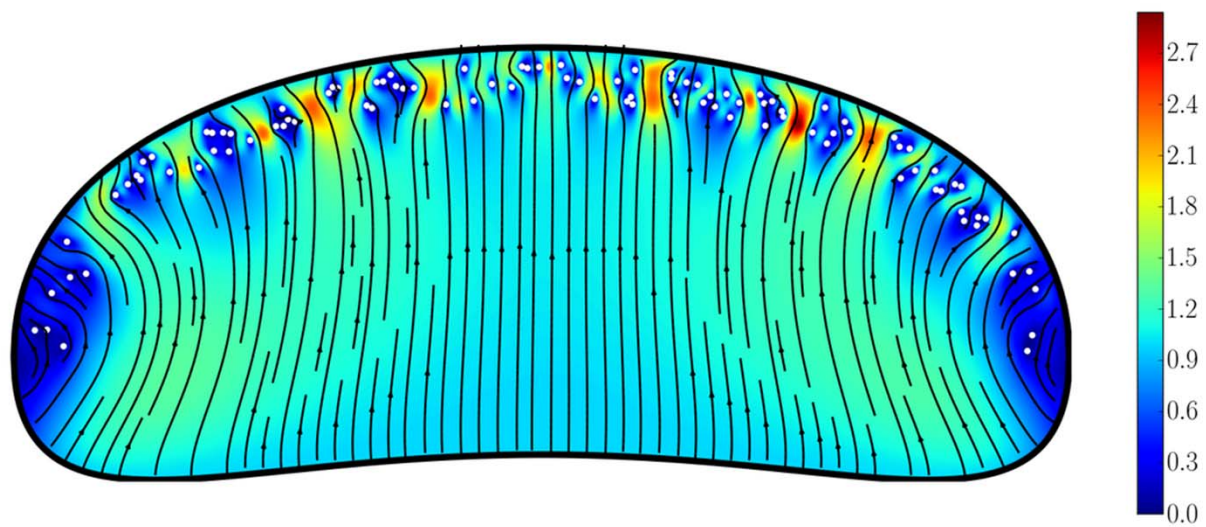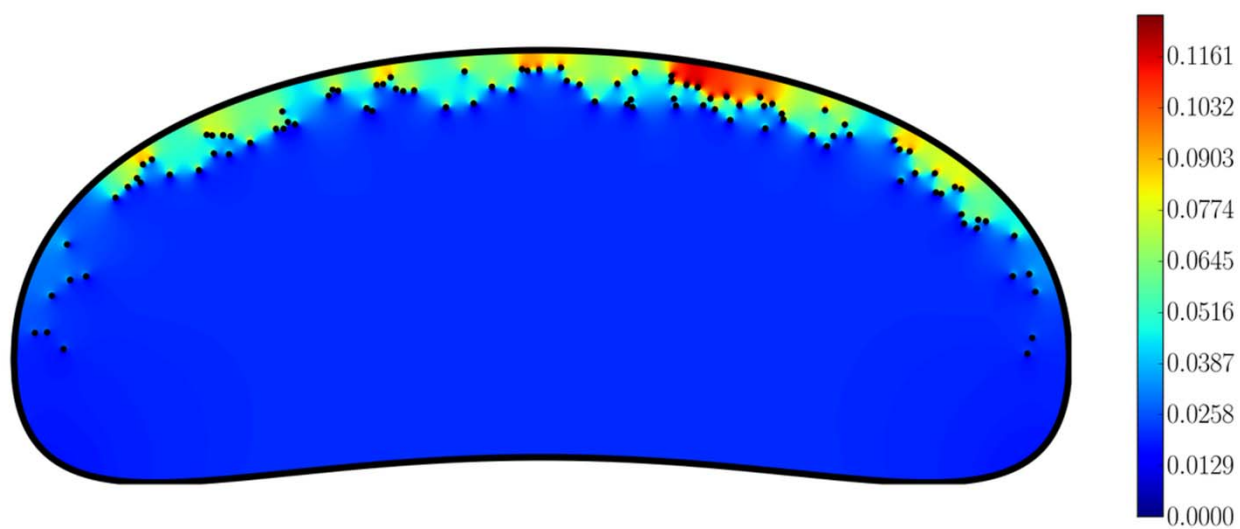

Supplement: Figure S5 — Computed membrane velocity (top) and tension (bottom) for 100 front-loaded proteins of diameter 0.1 µm. Proteins are shown in white in the top figure and black in the bottom figure. (PDF) [file pone.0084524.s005.pdf]

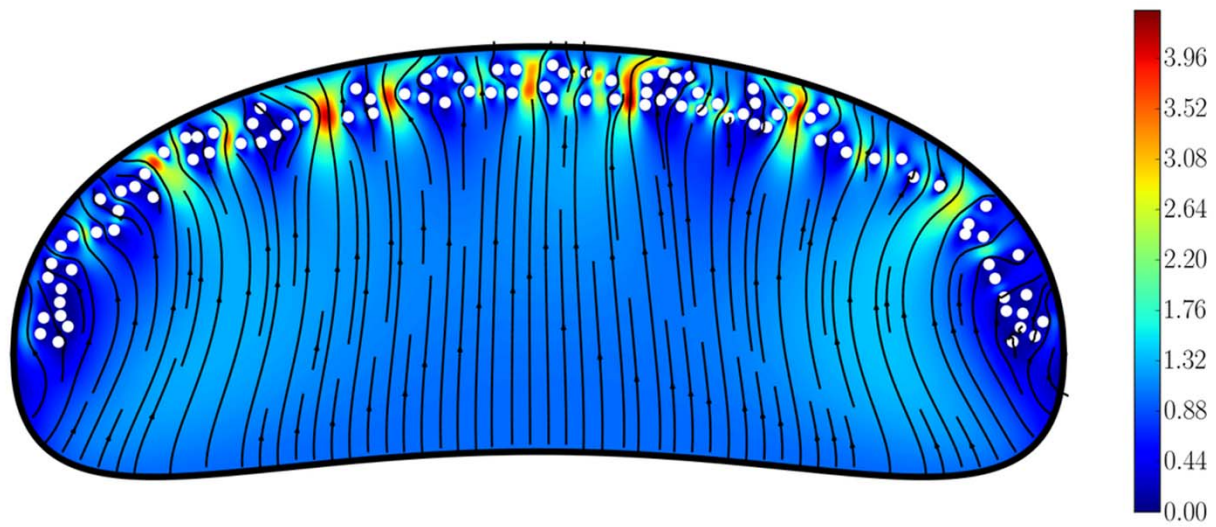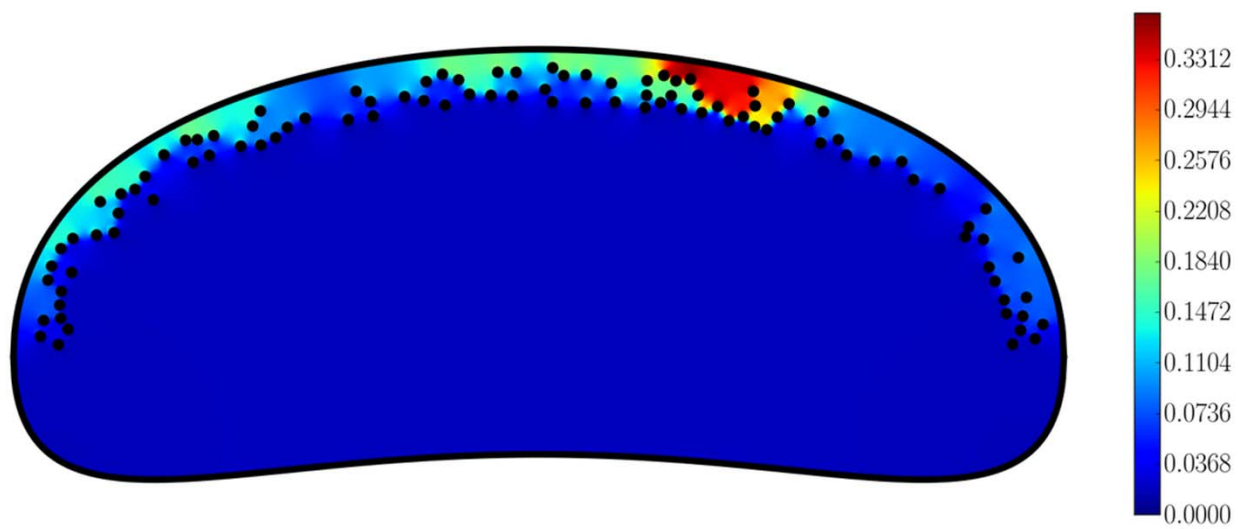

Supplement: Figure S6 — Computed membrane velocity (top) and tension (bottom) for 100 front-loaded proteins of diameter 0.2 µm. Proteins are shown in white in the top figure and black in the bottom figure. (PDF) [file pone.0084524.s006.pdf]

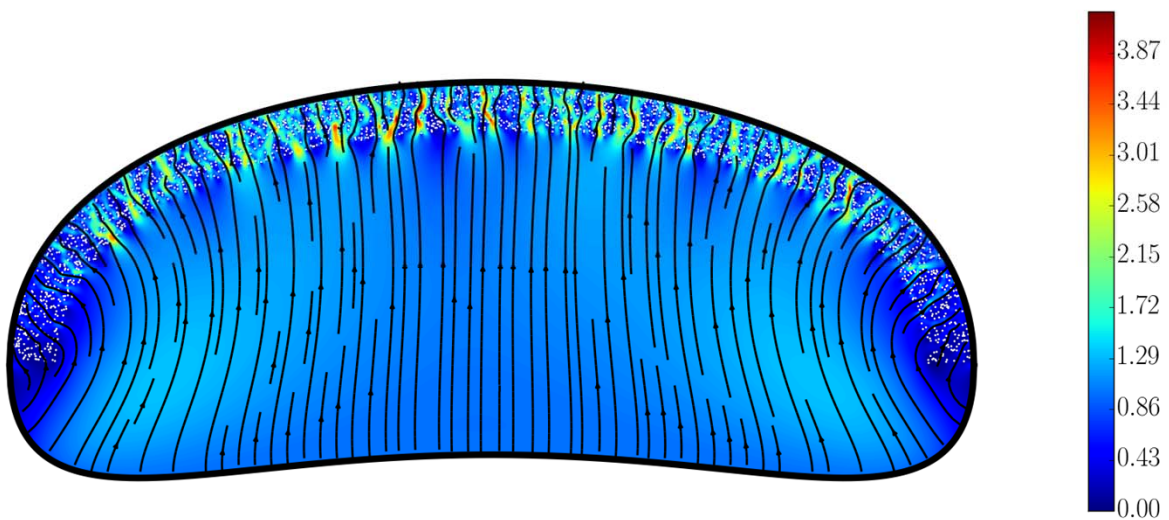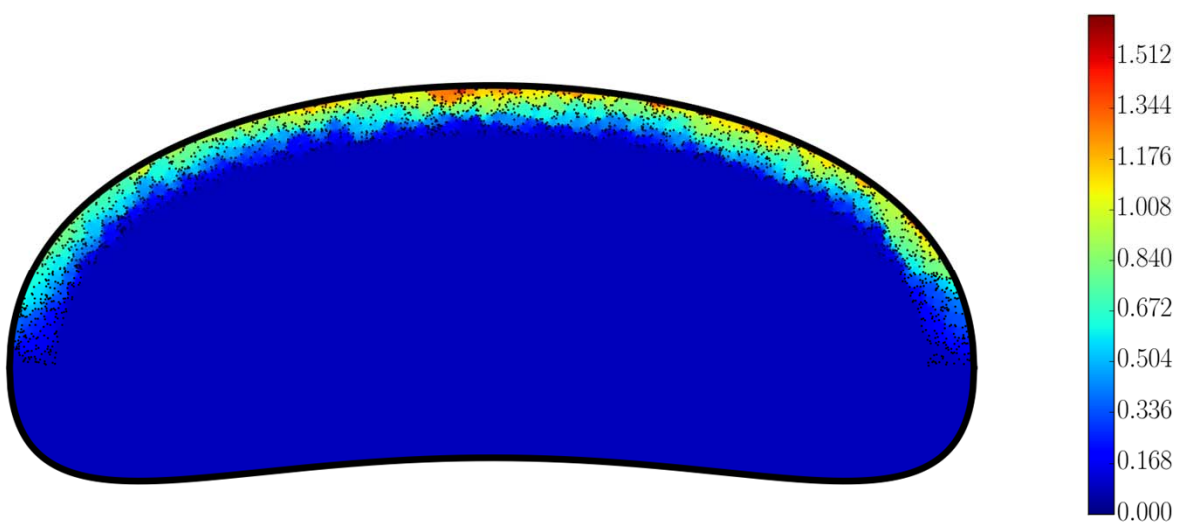

Supplement: Figure S7 — Computed membrane velocity (top) and tension (bottom) for 2000 front-loaded proteins of diameter 0.02 µm. (PDF) [file pone.0084524.s007.pdf]

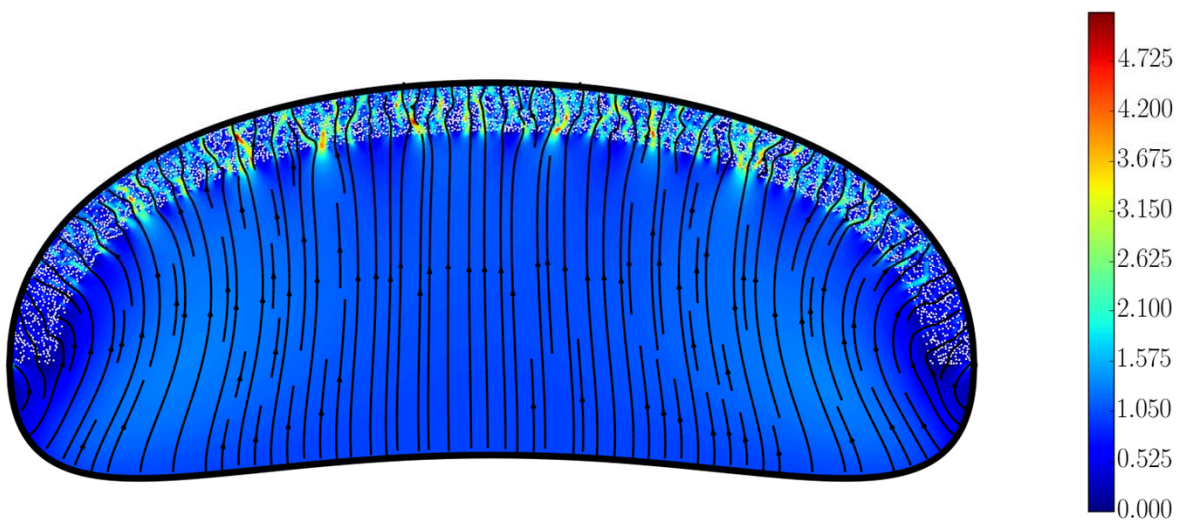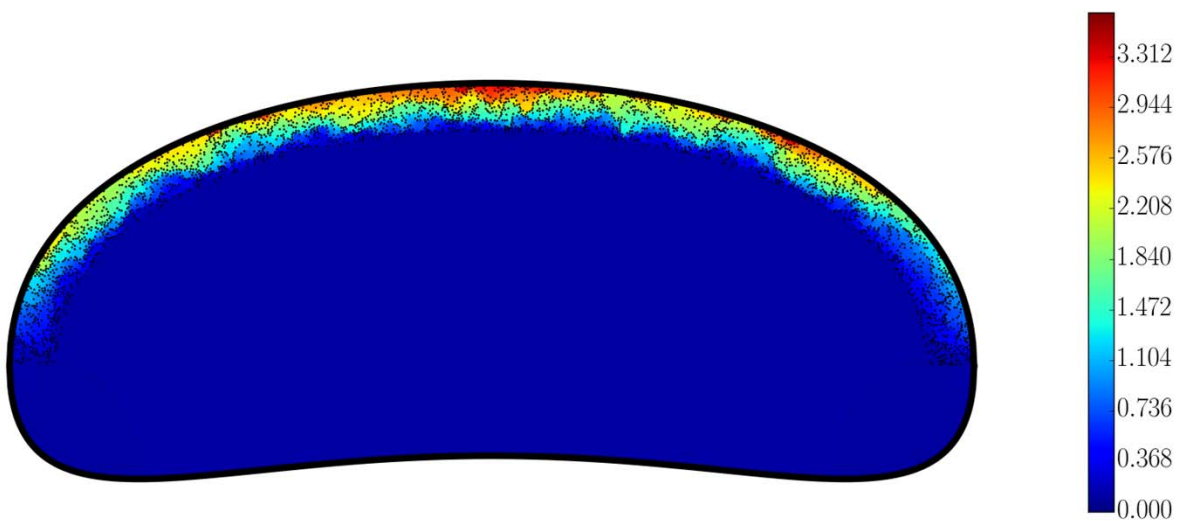

Supplement: Figure S8 — Computed membrane velocity (top) and tension (bottom) for 3000 front-loaded proteins of diameter 0.02 µm. (PDF) [file pone.0084524.s008.pdf]

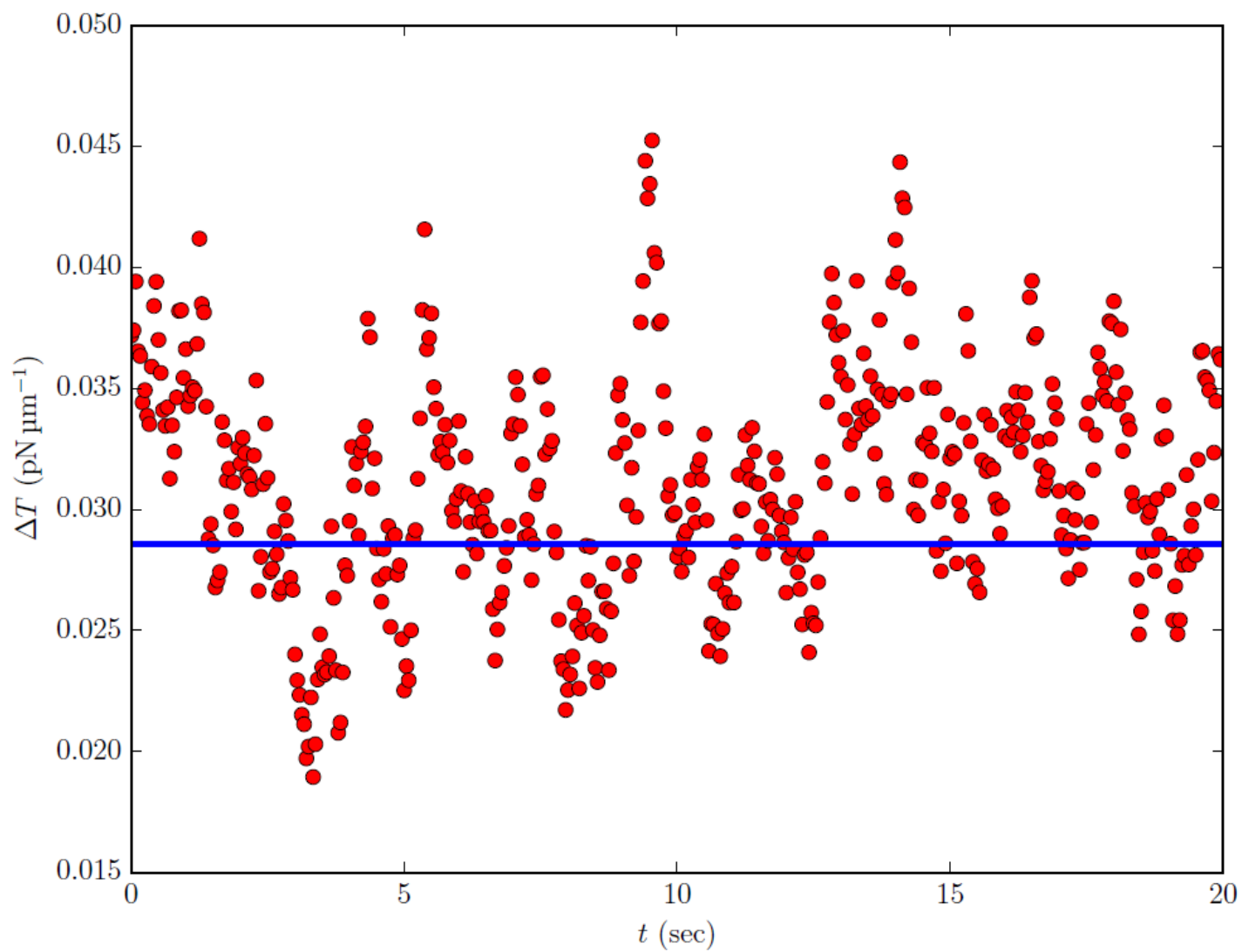

Supplement: Figure S9 — The membrane tension drop as a function of time. The membrane tension drop over the cell as a function of time (red), and the predicted tension drop from the analytical fit for this cell (blue), computed from the average values in the simulation of the moving cell over 200 seconds of simulated time. About 100 front-loaded proteins of diameter 0.1 µm are stationary, appear at the leading edge and disappear at the rear edge of the protein-loaded band, while the cell moves forward. (PDF) [file pone.0084524.s009.pdf]

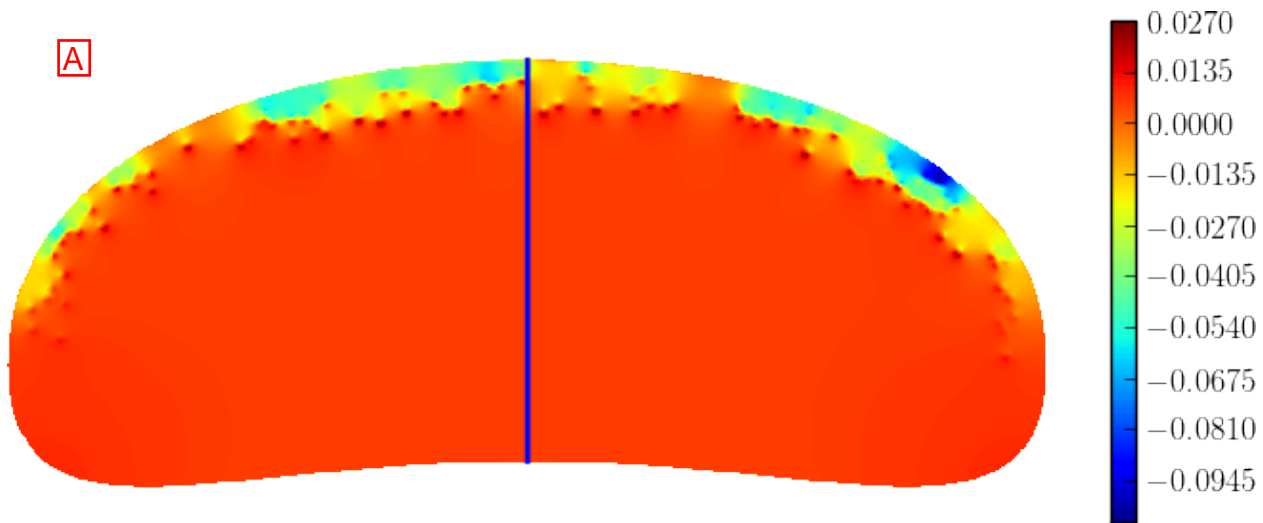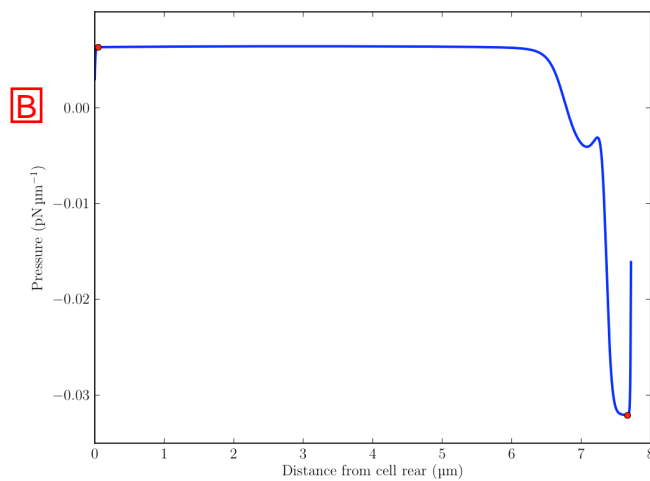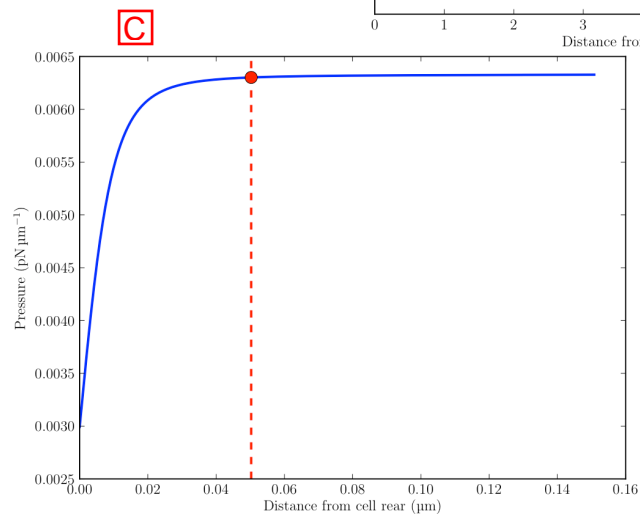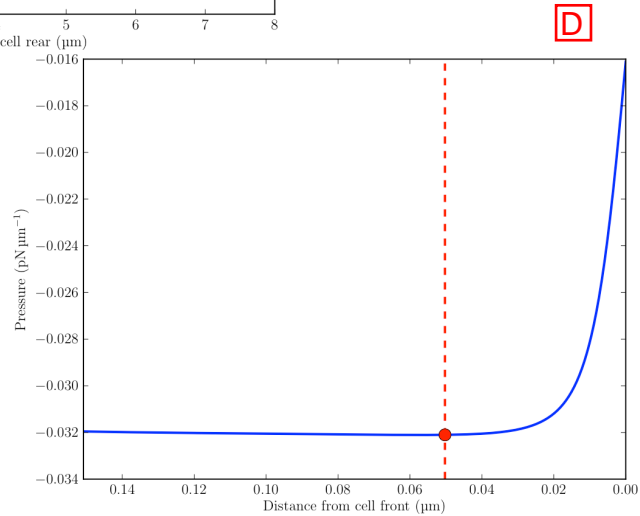

Supplement: Figure S10 — Pressure near cell boundaries. A: Pressure (pN/µm) in a simulated cell, with blue line through middle of the cell. B: Cross section plot showing pressure in cell along the cell's midline. Note that the pressure jumps sharply near both the rear and leading edges. C–D: Close up of the pressure cross section near the rear (C) and front (D) of the cell. Note the sharp jumps in pressure over very small spatial scales. In red (dots and dashed line), we show the position 4s≈0.05 µm away from the edges used for actual computation of edge pressures. Note also that membrane tension is equivalent to the pressure with the minus sign. (PDF) [file pone.0084524.s010.pdf]
